# Sporadic Parkinson’s Disease Potential Risk Loci Identified in Han Ancestry of Chinese Mainland

**DOI:** 10.3389/fnagi.2020.603793

**Published:** 2021-01-12

**Authors:** Bo Wang, Xin Liu, Shengyuan Xu, Zheng Liu, Yu Zhu, Xiong Zhang, Renshi Xu

**Affiliations:** ^1^Department of Neurology, The First Affiliated Hospital of Nanchang University, Nanchang, China; ^2^Department of Neurology, Jiangxi Provincial People’s Hospital, The Affiliated People’s Hospital of Nanchang University, Nanchang, China; ^3^Department of Neurology, First Affiliated Hospital of Gannan Medical University, Ganzhou, China; ^4^Department of Neurology, Maoming People’s Hospital, Maoming, China

**Keywords:** genetic polymorphism, single nucleotide polymorphisms, pathogenesis, sporadic Parkinson’s disease, Chinese Han ancestry

## Abstract

Recent investigations demonstrated that genetic factors might play an important role in sporadic Parkinson’s disease (sPD). To clarify the specific loci susceptibility to sPD, we analyze the relationship between 30 candidate single nucleotide polymorphisms (SNPs) and sPD in the population of Han ancestry from Chinese mainland (HACM) by using genome-wide association study, sequenom massARRAY, DNA sequence, and biological information analysis. Results showed that the subjects carrying the T allele of rs863108 and rs28499371 exhibited a decreased risk for sPD. The subjects carrying the T allele of rs80315856 exhibited an increased risk for sPD. The A/T genotype of rs863108 and the C/T genotype of rs28499371 were a potential increased risk for sPD, and the G/T genotype of rs80315856 and T/T genotype of rs2270568 were a potential decreased risk for sPD. The minor allele frequency (MAF) of rs80315856 and rs2270568 was higher in sPD. The T allele of rs80315856 and rs2270568 might be a risk locus for sPD. Our data suggested that the alteration of these SNPs might play some roles through changing/affecting LINC01524/LOC105372666, DMRT2/SMARCA2, PLEKHN1, and FLJ23172/FNDC3B genes in the pathogenesis of sPD.

## Introduction

Sporadic Parkinson’s disease (sPD) is a progressive neurodegenerative disease, which is characterized by signs and symptoms of progressive motor and non-motor dysfunctions. Due to the presence of Lewy bodies in dopamine (DA) neuron in the substantia nigra of the midbrain, the typical pathological features of sPD mainly display the progressive loss of DA neurons in the substantia nigra of the midbrain. The degenerated DA neurons in sPD primarily occur in the substantia nigra pars compacta (SNc) projected to striatum. sPD is one of commonest neurodegenerative diseases and occupies approximately 1% in the populations of more than 65 years (Marsden, 1994).

The characterized clinical manifestations of sPD include motor and non-motor symptoms. The typical clinical motor symptoms of sPD mainly consist of rest tremors, rigidity, bradykinesia, and posture disorder (Fahn, 2003; Cummings et al., 2011). The motor symptoms of sPD begin to manifest only after more than 50% dopaminergic neurons loss in SNc and a significant deficit of 70–80% striatal DA concentration in striatum. Besides motor manifestations, sPD reveals lots of non-motor symptoms. The commonest non-motor symptoms include anxiety, depression, olfactory hyposmia, the dysfunction of autonomic nerves (e.g., orthostatic symptoms, urine incontinence, and constipation), dyssomnia, behavioral disorders, and cognitive dysfunction (Göttlich et al., 2013; Khoo et al., 2013). sPD is a complex, multifactorial disease that can have diverse genetic, biological, and environmental influences (Polito et al., 2016). Although sPD patients lack evidential family history and definitive genetic basis, the familial forms of Parkinson’s disease (PD) have inferred that sPD might be closely associated with some genetic factors (Singleton et al., 2013). Up to now the etiology of sPD is still unclear, but it is convincing that the interaction of genetic factor, environmental factor, and aging together contributes to this disease (Kalia and Lang, 2015).

Over last two decades, we have witnessed a revolution in the field of sPD genetics. Great advances have been made in identifying many single nucleotide polymorphisms (SNPs) that confer a risk for sPD, which has subsequently led to an improved understanding of molecular pathways involved in the sPD pathogenesis. Despite this success, it is predicted that only relatively small proportion of phenotypic variability has been explained by genetics. Therefore, the heritable components of sPD are still waiting to be identified, and exploring the genetic architecture of sPD constitutes a critical effort in identifying the therapeutic targets of sPD. Although substantial progress has helped us to better understand the mechanism of sPD, the route to sPD-disease-modifying drugs is a lengthy one (Billingsley et al., 2018).

Although genetic and environmental factors currently have been shown to lead to sPD, the etiology of sPD remains elusive (Fahn, 2003). On the past unremitting effort, researchers have shed light on the relationship between some candidate genetic factors and sPD in different populations (von Otter et al., 2014; Rozenkrantz et al., 2016; Soldner et al., 2016; Wang et al., 2016; Yang et al., 2016). These genetic factors included SNPs, rearrangements, and insertion/deletion polymorphisms; however, very few studies have been evaluated in Chinese populations, especially in the Han ancestry of Chinese mainland (HACM).

To this end, in this study, we chose 30 most possible associated candidate SNPs from the results of our and other related previous study (Hu et al., 2016) to further analyze their susceptibility in 398 sPD patients and 430 controls from HACM. We discovered that the polymorphisms of rs863108, rs80315856, rs28499371, and rs2270568 were possibly associated with the pathogenesis of sPD from HACM. Furthermore, we also explored their potential biological functions and relationships in the pathogenesis of sPD. Our results suggested that the subject carrying the T allele (A/T + T/T) of rs863108 and the T allele (C/T + T/T) of rs28499371 exhibited a decreased risk for sPD. The subject carrying the T allele (T/T + G/T) of rs80315856 exhibited an increased risk for sPD. The A/T genotype of rs863108 and the C/T genotype of rs28499371 were a potential increased risk for sPD, and the G/T genotype of rs80315856 and the T/T genotype of rs2270568 were a potential decreased risk for sPD. The minor allele frequency (MAF) of rs80315856 was higher in sPD patients (8%) than in controls (6%). The MAF of rs2270568 was higher in sPD patients (47%) than in the controls (43%). The T allele of rs80315856 and rs2270568 may be the risk loci for sPD. These polymorphisms alteration might change or affect the structures and functions of LINC01524/LOC105372666, DMRT2/SMARCA2, PLEKHN1, and FLJ23172/FNDC3B genes to exert some effects in the pathogenesis of sPD.

## Materials and Methods

### Human Subjects

sPD and control dataset was created from two affiliated hospitals of the university, The First Affiliated Hospital of Nanchang University and The Affiliated Guangdong General Hospital of Nan Fang Medical University. All recruited sPD and control subjects were from HACM in Chinese southern regions (Jiangxi and Guangdong Province). Informed consent was signed by all participants in this study. All sPD patients were diagnosed according to clinical diagnostic criteria for sPD in China (Li et al., 2017). All subjects underwent the same evaluation process including records of medical history, Mini-Mental State Exam, PD family history, related diseases in their first-degree relatives, the toxicant exposure associated with sPD, related indispensable biochemical test, and brain and spinal magnetic resonance imaging to eliminate other neurological diseases that might mimic the clinical image of PD (e.g., tumors, demyelinated disorders, cerebral vascular disease, and Parkinsonism).

Studied populations were composed of 648 sPD and 680 controls. Among them, 250 sPD and 250 controls were used for genome-wide association study (GWAS) analysis (Hu et al., 2016); 398 sPD and 430 controls were used for the polymorphism analysis of 30 most possible associated candidate SNPs (Tables 1, 2). The male and female ratio of sPD was 215 (54.02%) and 183 (45.98%), and that of controls were 261 (60.69%) and 169 (39.31%). The mean (range) age of sPD was 60.99 ± 0.56 years, and that of controls were 62.06 ± 0.60 years (Table 1). Male subjects were more than female subjects because sPD affected more men than women, and our data exhibited a slight gender disparity. The age of the control subjects was older than that of sPD patients, which aimed to minimize the age bias that the control subjects were too young to develop sPD. Moreover, we also intend to avoid the age-related factors associated with sPD. For example, the sPD patients of early and late onset would be eliminated. The disease course of sPD patients was controlled within 3–5 years after onset, which would preclude the sPD patients of rapid and slow progression. The sPD patients with atypical clinical manifestations were eliminated yet. Based on the above enrolled factors of subjects, therefore, in our study, the sPD patients with typical age, clinical course, and phenotype were enrolled. This study was approved by the Institutional Review Board of the Hospital Human Ethics Committee of The First Affiliated Hospital of Nanchang University and The Affiliated Guangdong General Hospital of Nan Fang Medical University.

**TABLE 1:** Basic characteristics of study subjects.

	**Cases (*n* = 398)**	**Controls (*n* = 430)**	***P*-value**
Male [n (%)]	215 (54.02)	261 (60.69)	
Female [n (%)]	183 (45.98)	169 (39.31)	0.052
Age (years)	60.99 ± 0.56	62.06 ± 0.60	0.199

*n, number.*

**TABLE 2:** All SNPs were chosen in the association study.

**Our previous pooling GWAS scanning SNPs**						**Function region SNPs and validated hot SNPs**			
	
**Gene**	**Chro**	**SNP ID**	**Position**	**OR (95%CI)**	***P*-value**	**SNP ID**	**Position**	**Function region**	**MAF in CHP**
TSG1/MANEA	6	rs9445283	95,187,922	8.2 (5.70–11.78)	1.37461E-27				
PDE10A	6	rs880121	166,068,329	3.72 (2.75–5.04)	4.47044E-17				
MDGA2	14	rs9323124	47,466,177	3.97 (2.91–5.42)	3.44125E-19	rsl2590500^*a,b*^	46,957,434	Missense	0.073
ATPBD4/LOC100288892	15	rsl7534343	36,296,150	4.30 (3.16–5.85)	7.4025E-16				
ZFP64/TSHZ2	20	rs863108	51,255,111	6.59 (4.2–10.20)	4.98978E-13				
PAQR3/ARD1B	4	rs201453169	80,119,145	0.18 (0.12–0.25)	2.15365E-14				
FLJ23172/FNDC3B	3	rs73180248	171,699,046	3.97 (2.82–5.60)	1.35108E-05	rs2270568^*a*^	172,329,071	Synonymous	0.476
CYPlBl/C2orf58	2	rs163090	38,313,632	0.36 (0.26–0.50)	5.42354E-09				
ANXA1/LOC100132423	9	rs10746953	76,917,840	2.62 (1.91–3.60)	5.35053E-09				
FLJ35379	13	rs61959631	76,477,028	2.37 (1.78–3.14)	7.34753E-09	rs7652177^*a,d*^	172,251,287	Missense	0.39
PLEKHN1	1	rs3829740	909,238	2.63 (1.91–3.63)	1.47259E-08	rs28499371^*a*^	966,748	Missense	0.131
						rs3829738^*a*^	973,929	Missense	0.248
DMRT2/SMARCA2	9	rs80315856	1,261,774	0.29 (0.19–0.44)	1.1753E-08	rs2279984^*a*^	1,048,496	Promoter	0.417
						rsl2002058^*a*^	1048,945	Promoter	0.136
ZNF396/IN080C	18	rsl362858	32,986,600	2.68 (1.92–3.73)	3.62598E-05				
C3orf67/LOC339902	3	rs6783485	59,427,797	0.20 (0.12–0.35)	2.05182E-08				
LOC285194/IGSF11	3	rsl879553	118,615,463	2.83 (1.98–4.04)	3.28729E-08				
FGF10/MRPS30	5	rsl3153459	44,515,935	2.45 (1.79–3.34)	3.17573E-08				
BARX1/PTPDC1	9	rsl0993010	96,749,048	2.29 (1.72–3.06)	6.5853E-08	rsll793856^*a*^	93,952,209	Synonymous	0.073
						sl91789925^*a*^	93,955,005	Missense	0.092
COL5A2	2	rslll86	189,897,394	2.79 (1.94–4.03)	1.37461E-27	rsl0197596^*a,c*^	189,039,507	Synonymous	0.117
						rs6434312^*a,c*^	189,043,211	Synonymous	0.107
						rsll691604^*a*^	189,180,039	Promoter	0.286

*Chro, Chromosome; SNP, Single nucleotide polymorphism; GWAS, Genome wide association study; OR, Odds ratio; CI, Confidence interval; MD, Missing Data; CHP, Chinese Han populations. ^*a*^Hu et al. (2016). ^*b*^Hellquist et al. (2009). ^*c*^Li et al. (2014). ^*d*^Allen et al. (2010). The underlined values indicates position in chromosome.*

### Selection of SNPs

Based on the information of candidate genes and the purpose of this study, the screen scheme of candidate SNPs initially was formulated. In our previous pooling GWAS of 250 sPD patients and 250 control subjects from HACM, we revealed that 18 SNPs, including rs9445283 (kgp154172) in TSG1/MANEA, rs880121 (kgp8130520) in PDE10A, rs9323124 in MDGA2, rs17534343 (kgp11333367) in ATPBD4/LOC100288892, rs863108 (kgp4156164) in ZFP64/TSHZ2, rs201453169 (kgp9482779) in PAQR3/ARD1B, rs73180248 (kgp760898) in FLJ23172/FNDC3B, rs163090 (kgp11353523) in CYP1B1/C2orf58, rs10746953 in ANXA1/LOC100130911, rs61959631 (kgp9550589) in FLJ35379/LOC100132423, rs3829740 (kgp7172368) in PLEKHN1, rs80315856 (kgp10769919) in DMRT2/SMARCA2, rs1362858 in ZNF396/INO80C, rs6783485 in C3orf67/LOC339902, rs1879553 in LOC285194/IGSF11, rs13153459 in FGF10/MRPS30, rs10993010 (kgp6542803) in BARX1/PTPDC1, and rs11186 in COL5 A2, were strongly associated with sPD from HACM because their significance threshold was *p* < 3.7 × 10^–5^ (Hu et al., 2016). The above SNPs were not reported to be associated with sPD before our study (Hu et al., 2016). Moreover, we screened the previously reported candidate SNPs related with the pathogenesis of sPD by the screen methods of function region SNPs and validated hot SNPs (Supplementary Table 1). We found that 12 SNPs, which consisted of rs12590500 in MDGA2 (Hellquist et al., 2009; Hu et al., 2016); rs28499371 and rs3829738 in PLEKHN1 (Hu et al., 2016); rs10197596, rs6434312 (Li et al., 2014; Hu et al., 2016), and rs11691604 in COL5A2 (Hu et al., 2016); rs11793856 and rs191789925 in BARX1 (Hu et al., 2016); rs2279984 and rs12002058 in DMRT2 (Hu et al., 2016); and rs7652177 (Allen et al., 2010; Hu et al., 2016) and rs2270568 in FLJ23172/FNDC3B (Hu et al., 2016) were the possible susceptible SNPs of sPD based on the result of function region SNPs and validated hot SNPs screen (Supplementary Table 1). In addition, 12 additional SNPs were selected so as to (a) be within the wider loci indicated by the previous GWAS in Han Chinese and (b) reside within functional regions. Therefore, in this study, 30 SNPs were chosen to further ascertain their association with the pathogenesis of sPD (Table 2).

### Screen Methods of Function Region SNPs and Validated Hot SNPs Screen of Function Region SNPs

The functional SNPs screening of MAF > 0.025 in the Chinese Han population was conducted in two databases of HapMap^1^ and 1000 Genomes^2^ in exons, 5′ untranslated region (UTR), promoter, and 3′ UTR regions of candidate gene SNPs in the Geneview of National Center for Biotechnology Information (NCBI) website, which further verified the researched state in the relevant literature. The function of selected SNPs was predicted through http://snpinfo.niehs.nih.gov/, and their functions stated in some documents were also tagged.

### Screen of Validated Hot SNPs

Through searching the literature in the PubMed of NCBI and Google, we carried out the literature research of related SNPs of candidate genes and screened the susceptible SNPs. Screened SNPs were verified by MAF in the Chinese Han populations in the HapMap and 1000 Genomes database (see text footnotes 1, 2). SNPs of MAF < 0.025 is abandoned.

### SNPs Genotype of Sequenom Mass Array

SNPs genotype was performed by sequenom massARRAY platform (Sequenom, San Diego, CA, United States) according to manufacturer’s instructions. Thirty SNPs were genotyped. For quality control, 5% of the samples underwent repeated genotype; 100% of the results was consistent. This experiment was conducted by Wuhan Icongene Biological Technology Co., Ltd (Contract number: BMSW20170807LHY0063).

### Major Reagents and Apparatuses

Major reagents used in the experiments were the following: DNA extraction kit (BioTeKe Corpration), HotStar Taq (5 U/μl), shrimp alkaline phosphatase (SAP) buffer, SAP enzyme, iPLEX buffer plus, iPLEX termination mix, iPLEX enzyme, and massARRAY TYPER4.0 (Agena, Inc.). The main apparatuses applied for this research were as follows: nucleic acid automatic extractor (BioTeKe Corpration), nanoDrop2000 (Thermo Fisher Scientific, United States), Veriti-384 PCR reactor (ABI, United States), 384-well spectroCHIP bioarray chip^®^, massARRAY nanodispenser spotting machine, and massARRAY analyzer 4.0 mass spectrometer (Agena, Inc.).

### Performance of 384-Well PCR Reactions

DNA assays were conducted by 384-well PCR reactions in this study. The experimental processes were as follows: (1) a PCR mixture was prepared with the following descriptions—1.85 μl ddH_2_O, 0.625 μl 10 × PCR buffer with 15 mM MgCl_2_, 0.325 μl 25 mM MgCl_2_, 0.1 μl deoxyribonucleotide triphosphate (dNTP) mix (25 mM), 0.1 μl HotStar Taq PCR enzyme (5 U/μl), 1 μl primer mixture (0.5 μM), 1 μl DNA template (20 ng/μl), a total of 5 μl volume; (2) 5 μl PCR mixture was added to each well of 384-well microtiter plate; (3) the microtiter plate was centrifuged at 1,000 rpm for 1 min; and (4) the 384-well microtiter plate was thermocycled in the following amplified conditions—94°C degeneration for 20 s, 56°C annealing for 30 s, and 72°C extension for 1 min, a total of 45 cycles. The primers in Supplementary Table 2 were used in the above 384-well PCR reactions.

### Preparation of SAP Enzyme Solution

The SAP enzyme solution was prepared with the following descriptions: mixed 1.53 μl RNase-free ddH_2_O, 0.3 μl 1 U/μl SAP enzyme, and 0.17 μl 10 × SAP buffer in a 2-μl tube; held the 1.5-mL tube containing the SAP enzyme solution onto a vortex to mix the solution for 5 s; and centrifuged the SAP enzyme solution at 5,000 rpm for 10 s.

### Treatment of PCR Production by SAP Enzyme

Two microliters SAP enzyme solution was added into each well of 384-well sample microtiter plate. The 384-well sample microtiter plate was sealed using a plate sealing film, centrifuged at 1,000 rpm for 1 min, and incubated as follows: 37°C for 20 min and 85°C for 5 min.

### Preparation of High Plex iPLEX Gold Reaction Mixture

The high plex iPLEX gold reaction mixture was prepared as follows: 0.619 μl RNase-free ddH_2_O, 0.2 μl iPLEX buffer plus, 0.2 μl iPLEX termination mixture, 0.94 μl iPLEX extend primer mixture (7 μM/14 μM), 0.041 μl 1 × iPLEX enzyme, and 7 μl PCR/SAP reaction solution were added to a total of 9 μl volume; the mixture was centrifuged in a microtiter plate at 1,000 rpm for 1 min.

### High Plex iPLEX^®^ Gold Assay

The 2 μl high plex iPLEX gold reaction mixture was added into the 384-well sample microtiter plate. The 384-well sample microtiter plate was sealed with a plate sealing film, centrifuged at 1,000 rpm for 1 min, and thermocycled as follows: 94°C degeneration for 30 s (94°C degeneration for 5 s, 52°C annealing for 5 s, 80°C extension for 5 s, 5 cycles), 52°C annealing for 5 s, and 72°C extension for 3 min, a total of 40 cycles. The primers in Supplementary Table 2 were used.

### Cleanup of High Plex iPLEX Gold Reaction Products

The cleanup of high plex iPLEX gold reaction products involved adding water and cleaning. The processes performed were as follows: the sample microtiter plate was resined; the clean resin was spread onto a 384-well dimple plate; nanopure water was added into each well of the 384-well sample microtiter plate and then the clean resin was added; and the 384-well sample microtiter plate was rotated and centrifuged.

### Acquirement of Spectra

The ACQUIRE module controlled massARRAY analyzer compact was used to acquire spectra from SpectroCHIPs. Each SpectroCHIP spectra was processed and analyze by massARRAY TYPER 4.0 software.

### Sanger Sequence of DNA

For each SNPs, the Sanger sequence of samples subset was performed on an ABI3500 (ABI3730xl; Applied Biosystems, Inc., CA) to confirm the genotyping of sequenom massARRAY. Their nucleotide variants were analyzed with DNASTARLaser gene software (Version v7.1) and compared with DNA sequence from NCBI GeneBank. This experiment was performed by Wuhan Icongene Biological Technology Co., Ltd (Contract number: XM171114-471).

### Function Prediction of rs863108, rs80315856, rs28499371, and rs2270568

The functional prediction of polymorphisms in rs863108 of the LINC01524/LOC105372666 gene, rs80315856 of the DMRT2/SMARCA2 gene, rs28499371 of the PLEKHN1 gene, and rs2270568 of the FLJ23172/FNDC3B gene was further conducted, which included transcription factor binding sites (Alkema et al., 2004), pathogenicity, protein structure domain, protein family evolution, protein Q494U1 homology modeling, and secondary structure of protein Q53EP0. The transcription factor binding sites in the sequence 1,500 bp upstream in the mutation position was analyzed using Mscan^3^. Human messenger RNA (mRNA) sequences were downloaded from the Ensemble website of version GRCh38 and formatted as database. The sequence of mutation position was applied to Blast against the database. Blast with an e-value cutoff e-15 was performed to obtain hits with the database. The pathogenicity prediction of the mutation site was analyzed using polyphen database^4^ and sift database^5^. Pathogenicity screening was performed with a polyphen pathogenicity score cutoff of 0.8 and sift pathogenicity score cutoff of 0.05. Coding protein sequence was retrieved from uniprot database^6^. Protein structure domain was analyzed using NCBI CDD database with input sequence and default parameter. For protein family evolution analysis, an evolution tree was drawn by gene tree tool in the Ensemble database. Multiple alignment sequence was extracted from gene tree results and was applied to draw seqLogo figure using weblogo software^7^. Protein Q494U1 homology modeling was analyzed using I-Tasser software^8^ with *ab initio* algorithm. The modeling structures of the mutation and origin proteins were aligned using SuperPose software^9^. The secondary structure of protein Q53EP0 was retrieved by SWISS-MODEL database^10^; the protein secondary structure of mutation sites was exhibited in the structure.

### Statistical Analysis

Statistical analysis was performed using SPSS (Version 18.0) statistical software (SPSS, Chicago, IL, United States). Hardy–Weinberg equilibrium (HWE) was first evaluated in healthy controls. Pearson chi-square test was used to compare the frequency of allele and genotypes in both cases and controls. MAF and odds ratios (ORs) with 95% confidence intervals (CI) were estimated to determine the role of each SNPs in sPD patients and controls. Two-tailed *p* < 0.05 were considered as statistical significance.

## Results

### Identification of Genetic Association Between 30 SNPs and sPD

We analyzed the association of 30 SNPs (Table 2) and sPD in an independent Chinese population using sequenom massARRAY technology. Four novel SNPs of rs863108 in the LINC01524/LOC105372666 gene (Chr 20:52638572), rs80315856 in the DMRT2/SMARCA2 gene (Chr 9:1261774), rs28499371 in the PLEKHN1 gene (Chr 1:966748), and rs2270568 in the FLJ23172/FNDC3B gene (Chr 3:172329071) were identified in our study.

The allelic and genotypic frequencies are summarized in Tables 3, 4. Four novel SNPs were identified as follows: rs863108, rs80315856, rs28499371, and rs2270568. The subjects carrying the T allele (A/T + T/T) of rs863108 (*OR* = 0.73, 95% *CI* = 0.55–0.97, *p* = 0.029) and the subjects carrying T allele (C/T + T/T) of rs28499371 (*OR* = 0.75, 95% *CI* = 0.56–1.00, *p* = 0.05) exhibited a decreased risk for sPD in comparison with other subjects. The subjects carrying the T allele (T/T + G/T) of rs80315856 (*OR* = 1.51, 95% *CI* = 1.05–2.19, *p* = 0.027) exhibited an increased risk for sPD in comparison with other subjects. The subjects carrying the T allele (T/T + C/T) of rs2270568 (*OR* = 1.23, 95% *CI* = 0.94–1.61, *p* = 0.133) exhibited no significance in comparison with other subjects (Table 3). The A/T genotype of rs863108 and C/T genotype of rs28499371 were a potential increased risk for sPD, and the G/T genotype of rs80315856 and the T/T genotype of rs2270568 were a potential decreased risk for sPD compared with other genotypes (Table 3). The MAF of rs80315856 (*OR* = 1.460, 95% *CI* = 1.027–2.077, *p* = 0.034) was significantly different between sPD patients and controls. The MAF of rs80315856 was higher in sPD patients (8%) than in controls (6%). The MAF of rs2270568 (*OR* = 1.217, 95% *CI* = 1.020–1.452, *p* = 0.029) was significantly different between sPD patients and controls. The MAF of rs2270568 was higher in sPD patients (47%) than in controls (43%). The T allele of rs80315856 and rs2270568 may be the risk loci of sPD. The MAF of rs863108 and rs28499371 did not find any significant difference between sPD patients and controls (Table 4).

**TABLE 3:** Association between four SNPs and the risk of sPD in the Chinese Han population.

**SNP**	**Genotype**	**Cases**	**Controls**	**OR (95% CI)**	***P*-value**
rs863108	A/A	145 (0.29)	112 (0.23)	1	Ref
(Chr 20:52638572 T > A)	A/T	243 (0.48)	267 (0.54)	0.7 (0.52–0.95)	0.022
	T/T	114 (0.23)	112 (0.23)	0.79 (0.55–1.13)	0.188
	T/T + A/T	357	379	0.73 (0.55–0.97)	0.029
rs80315856	G/G	428 (0.842)	445 (0.89)	1	Ref
(Chr 9:1261774 G > T)	G/T	79 (0.156)	54 (0.108)	1.52 (1.05–2.20)	0.026
	T/T	1 (0.002)	1 (0.002)	1.04 (0.06–16.68)	1.00
	T/T + G/T	80	55	1.51 (1.05–2.19)	0.027
rs28499371	C/C	402 (0.79)	359 (0.74)	1	Ref
(Chr 1:966748 C > T)	C/T	96 (0.19)	119 (0.24)	0.72 (0.53–0.98)	0.034
	T/T	10 (0.02)	8 (0.02)	1.12 (0.44–2.86)	0.823
	T/T + C/T	106	127	0.75 (0.56–1.00)	0.050
rs2270568	C/C	141 (0.28)	158 (0.32)	1	Ref
(Chr 3:172329071 T > C)	C/T	252 (0.50)	249 (0.51)	1.13 (0.85–1.51)	0.390
	T/T	115 (0.22)	85 (0.17)	1.52 (1.06–2.17)	0.024
	T/T + C/T	367	334	1.23 (0.94–1.61)	0.133

*SNP, single nucleotide polymorphism; CI, confidence interval; OR, odds ratio; sPD, sporadic Parkinson’s disease; p significance at < 0.05. All p-values were calculated by means of chi-squared test.*

**TABLE 4:** Allele frequencies of four SNPs in sPD cases and controls groups.

**SNPs**	**Allele**	**Cases**	**Controls**	**χ^2^**	***P***	**OR (95% CI)**
rs80315856	G (Major)	935 (0.92)	944 (0.94)			0.685 (0.481–0.974)
	T (Minor)	81 (0.08)	56 (0.06)	4.478	0.034	1.460 (1.027–2.077)
rs2270568	C (Major)	534 (0.53)	565 (0.57)			0.822 (0.689–0.980)
	T (Minor)	482 (0.47)	419 (0.43)	4.769	0.029	1.217 (1.020–1.452)

*SNPs, single nucleotide polymorphism; CI, confidence interval; OR, odds ratio; sPD, sporadic Parkinson’s disease; χ^2^, chi-squared test. p significance at < 0.05. The data weren’t shown in this table, because the MAF of rs863108 and rs28499371 didn’t find any significant difference between sPD patients and controls (The p-value of χ^2^-test > 0.05).*

### Confirmation of Variation Position in rs863108, rs80315856, rs28499371, and rs2270568

**FIGURE 1:**
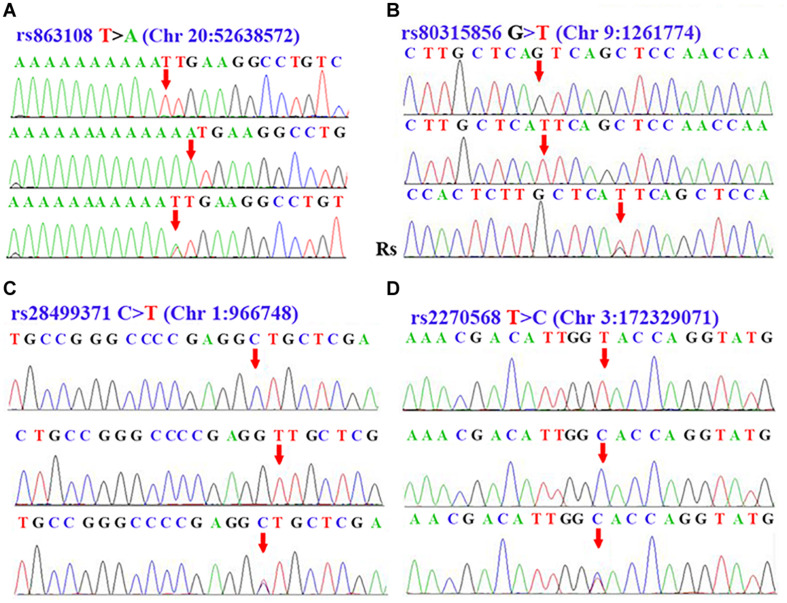
Sequence data of rs863108, rs80315856, rs28499371, and rs2270568. **(A)** Peak graph showing T > A variation of rs863108 in LINC01524/LOC105372666 gene. **(B)** Peak graph showing G > T variation of rs80315856 in DMRT2/SMARCA2. **(C)** Peak graph showing C > T variation of rs28499371 in PLEKHN1. **(D)** Peak graph showing T > C variation of rs2270568 in FLJ23172/FNDC3B. The variant loci were indicated by the red arrow. The Rs in the Panel **(B)** was the abbreviation of reverse sequence, which indicate that the Rs sequence in the Panel **(B)** was the result of reverse DNA sequence.

After performing a sequenom massARRAY array, we randomly drew 10 samples of each SNPs to reconfirm the genotypes of rs863108, rs80315856, rs28499371, and rs2270568 by conducting DNA Sanger sequence on an ABI3500. Sequencing results coincided with that from our sequenom massARRAY array. The polymorphism of rs863108 in the LINC01524/LOC105372666 gene was a T > A variation (Figure 1A). The polymorphism of rs80315856 in the DMRT2/SMARCA2 gene was a G > A variation (Figure 1B). The polymorphism of rs28499371 in the PLEKHN1 gene was a C > T variation (Figure 1C). The polymorphism of rs2270568 in the FLJ23172/FNDC3B gene was a T > C variation (Figure 1D and Table 3).

### General Mutation Information and Prediction of Transcription Factor Binding Site of rs863108

**FIGURE 2:**
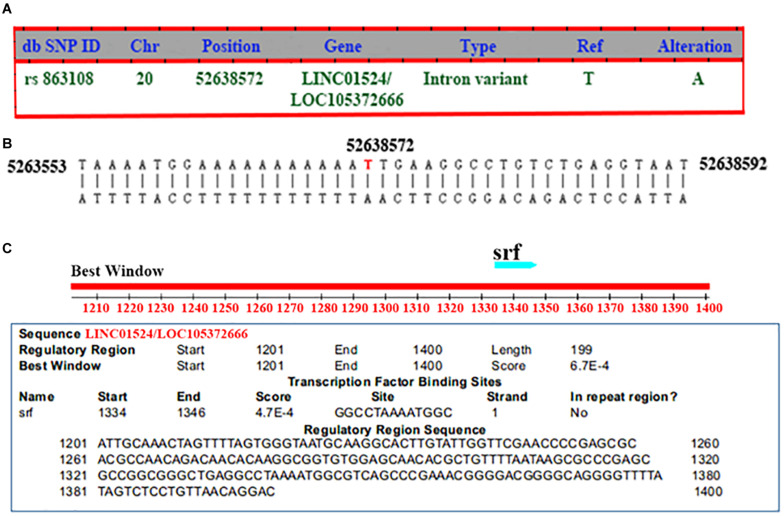
General mutation information and transcription factor binding site prediction of rs863108. **(A)** General mutation information of rs863108. The mutation site of rs863108 located in Chr20:52638572 position of LINC01524/LOC105372666 gene changed from T to A. **(B)** rs863018 mutation in the chromosome. The rs863018 mutation located in the hr20:52638572 chromosome position. **(C)** Prediction of transcription factor binding site. The transcription factor binding site was predicted in GGCCTAAAATGGC.

The mutation information of rs863108 (*Homo sapiens*) was retrieved from NCBI dbSNP database^11^ as shown in Figure 2A. The LINC01524/LOC105372666 mutation site of rs863108 located in the Chr20:52638572 changed from T to A as shown in Figure 2B. The transcription factor binding site was predicted at distances from 166 to 154 bp upstream of LINC01524/LOC105372666 as shown in Figure 2C.

### General Mutation Information, Pathogenicity Prediction, Analysis of Structure Domain, Protein Family Evolution, and Structure Modeling of rs28499371

**FIGURE 3:**
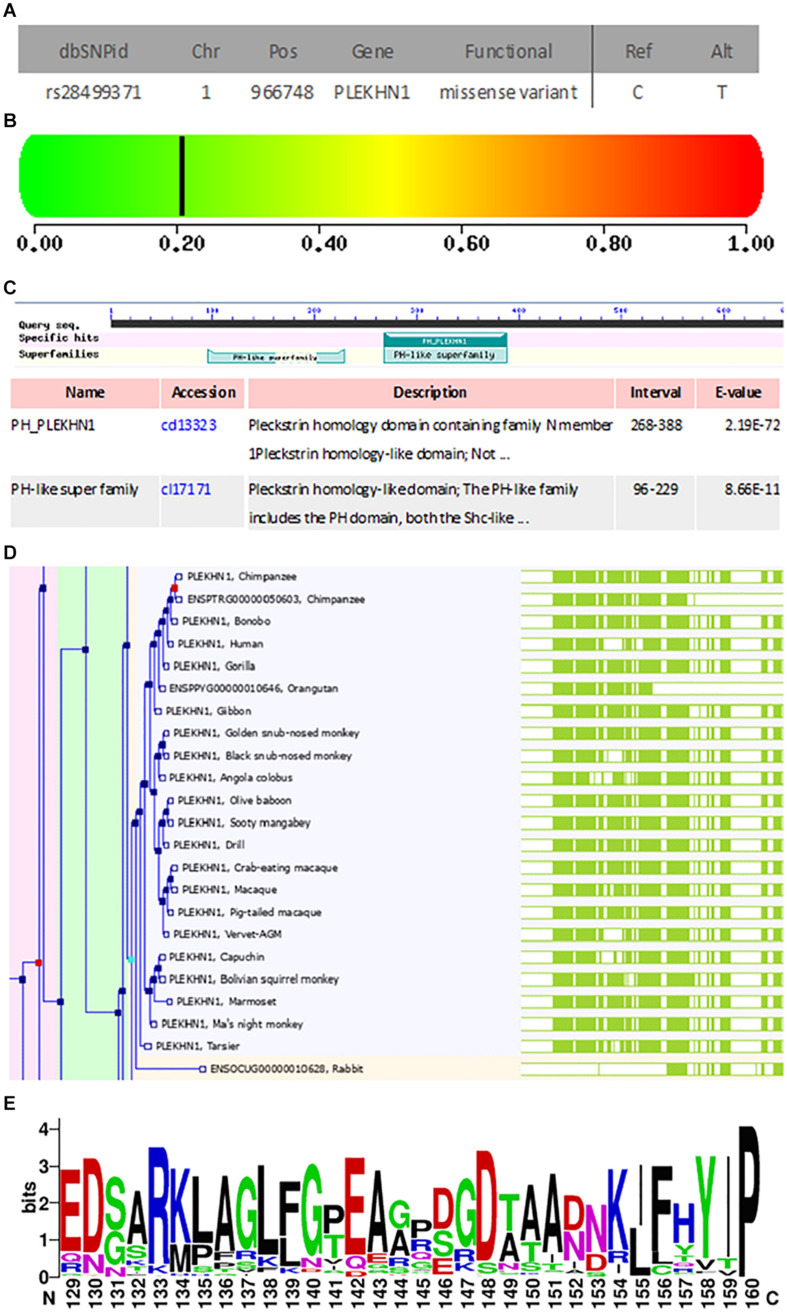
General mutation information, pathogenicity prediction, structure domain analysis, and protein family evolution analysis of rs28499371. **(A)** General mutation information. The mutation site located in the Chr1:966748 changed from C to T. This mutation is a missense variant, which leads to the codon change from GCT to GTT and the amino acid change from A to V in the 43rd position of the coding protein. **(B)** Pathogenicity prediction of A43V mutation site. This mutation is predicted to be benign with a score of 0.208 (sensitivity, 0.92; specificity, 0.88). **(C)** PLEKHN1 structure domain analysis. PLEKHN1 is the PH-like super family. **(D,E)** The mutation site was in the 43rd amino acid position and did not appear in the protein structure domain. Analysis of protein family evolution. **(D)** PLEKHN1 gene evolution tree. **(E)** PLEKHN1 weblogo. The mutation sites located in the 143rd amino acid position and the main mutations are A, E, G, and S. In those mutations, A is the most important, and V is rare. The V mutation may lead to the pathogenicity.

**FIGURE 4:**
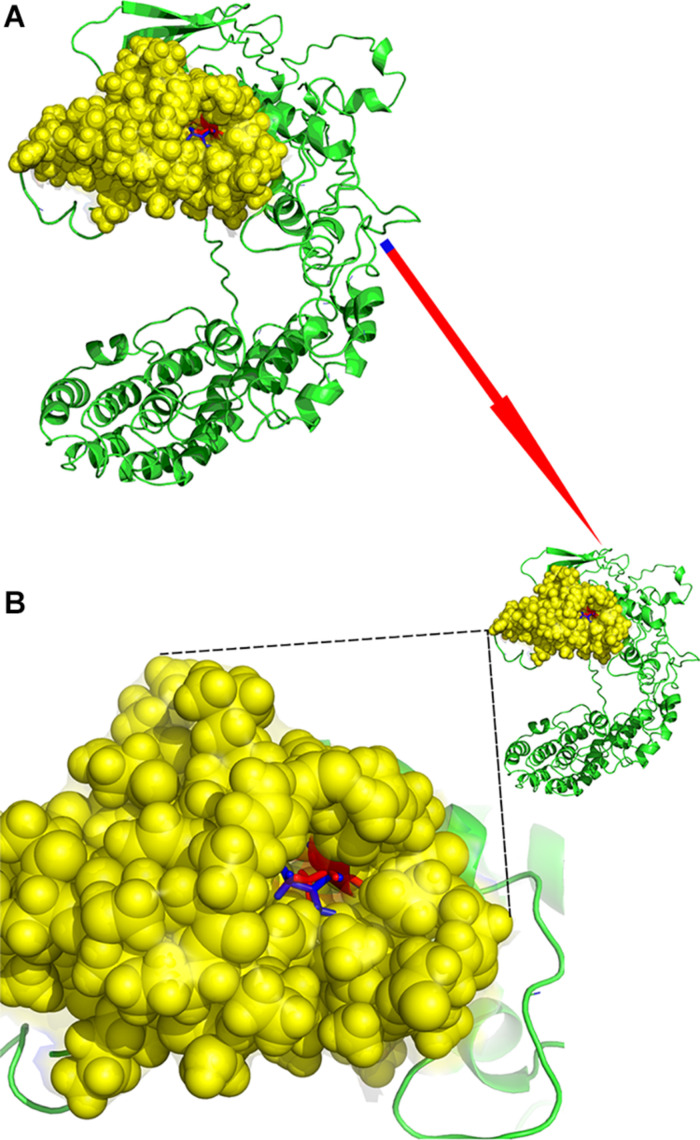
Structure modeling analysis of rs28499371. **(A)** Protein alignment. **(B)** Mutation structure. The neighborhood partial structure was retained. The mutation amino acid was located at the center of protein pocket, and this may influence the neighborhood structure. Red represents the origin protein structure; blue represents the mutation protein structure.

rs28499371 (*H. sapiens*) information was retrieved from NCBI dbSNP database (see text footnote 11) as shown in Figure 3A. The mutation site located in Chr1:966748 changed from C to T (Figure 3A). This mutation is missense variant, which leads to codon change from GCT to GTT and amino acid change from A to V in the 43rd position of PLEKHN1 coding protein (Figure 3A). The pathogenicity prediction of mutation site was conducted as shown in Figure 3B. This mutation was predicted to be benign with a score of 0.208 (sensitivity, 0.92; specificity, 0.88) (Figure 3B). The result showed that PLEKHN1 was PH-like super family in Figure 3C. The mutation site was in the 43rd amino acid position and did not appear in the protein structure domain (Figure 3C). The PLEKHN1 evolution tree was drawn by the gene tree tool in Ensemble database, and 80 orthologous genes were found in the database as shown in Figure 3D. Multiple alignment sequences were extracted from the gene tree result and were applied to draw seqlogo figure using weblogo software^12^ as shown in Figure 3E. The mutation sites located in the 143rd amino acid position and main mutations were A, E, G, and S. In those mutations, A is the most important, and V is rare. V mutation may lead to pathogenicity (Figures 3D,E). Structure modeling analysis was conducted. The modeling structures of mutation and origin proteins are showed in Figures 4A,B. In Figure 4, red represents origin protein structure, blue represents mutation protein structure. The neighborhood partial structure is retained in Figure 4A. The mutation amino acid is located at the center of protein pocket, which may influence neighborhood structure.

### General Mutation Information, Pathogenicity Prediction, and Structure Modeling of rs2270568 Mutation

**FIGURE 5:**
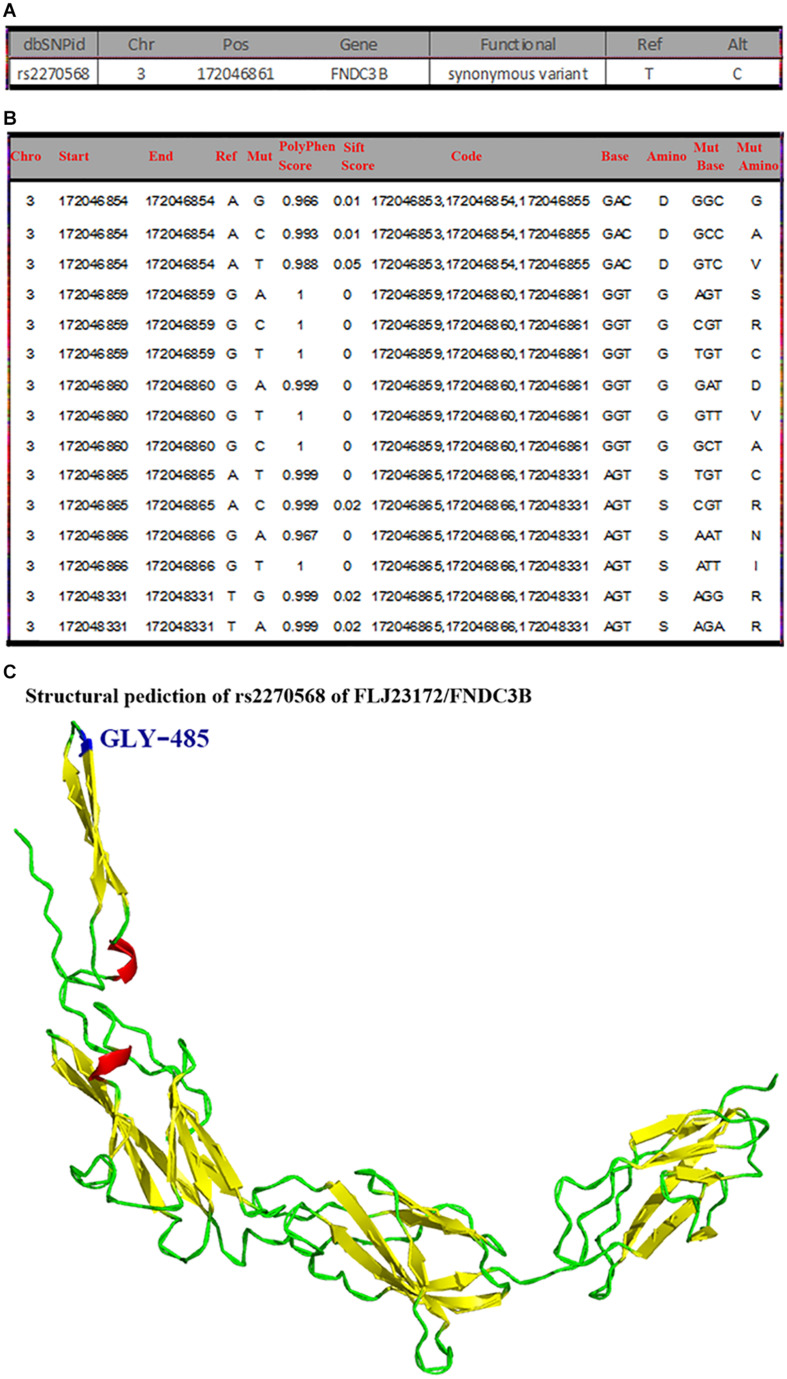
General mutation information, pathogenicity prediction, and structure modeling of rs2270568. **(A)** The mutation site of rs2270568 located in Chr3:172046861 position changed from T to C. **(B)** Multiple pathogenicity site near rs2270568. The 2,869 pathogenicity sites were screened by the databases, and the multiple pathogenicity sites were found near the rs2270568 mutation site as shown in the figure. These pathogenicity sites lead to the change in amino acid. **(C)** Structure modeling of mutation. The mutation site appeared between the coil and beta sheet, which were the pathogenicity mutation region with high frequency occurrence. The pathogenicity sites near the mutation site may influence the protein function and the disease regulation mechanism. However, this protein function may not be much influenced by the mutation site. Blue represents the mutation site, red represents the alpha helix, yellow represents the beta sheet, and green represents the random coil.

rs2270568 (*H. sapiens*) information was retrieved from NCBI dbSNP database (see text footnote 11) as shown in Figure 5A. The mutation site of rs2270568 located in Chr3:172046861 changed from T to C (Figure 5A). A total of 2,869 pathogenicity sites were screened by databases, and multiple pathogenicity sites were found near the rs2270568 mutation site as shown in Figure 5B. These pathogenicity sites led to the change in amino acid (Figure 5B). Mutation sites appeared between the coil and beta sheet, which were pathogenicity mutation regions with high frequency occurrence. The pathogenicity site near the mutation site may influence protein function and the regulation mechanism of disease (Figure 5C).

## Discussion

In this study, we found that four SNPs were possibly associated with sPD in HACM, rs863108 in the LINC01524/LOC105372666 gene, rs80315856 in the DMRT2/SMARCA2 gene (Hu et al., 2016), rs28499371 in the PLEKHN1 gene (Hu et al., 2016), and rs2270568 in the FLJ23172/FNDC3B gene (Hu et al., 2016). Among them, two SNPs of rs863108 and rs80315856 were our previously reported SNPs (Hu et al., 2016). This study further certified our past studied result (Hu et al., 2016).

LINC01524, long intergenic non-protein coding RNA 1524, locates in 20q13.2 and consists of 3 exons, which belongs to the non-coding RNA (ncRNA) gene type. LOC105372666 locates in 20q13.2 and consists of 18 exons, belonging to the ncRNA gene type. LINC01524 and LOC105372666 are mainly expressed in the human testis tissue (Strausberg et al., 2002; Fagerberg et al., 2014). In this study, we found that the T > A variation of rs863108 polymorphism in the LINC01524/LOC105372666 gene was possibly associated with the risk of sPD from HACM. At present, the relationship and pathogenesis about the LINC01524/LOC105372666 gene with sPD have not been reported. Based on the LINC01524/LOC105372666 gene mainly expressed in the human testis tissue that generate androgen, we hypothesized whether the pathogenesis of sPD was associated with the reduced secretion of androgen, as morbidity of sPD is higher in male than in female, and the partial sPD patients are accompanied with sexual dysfunction, which might be related with the abnormal expression of the LINC01524/LOC105372666 gene that contributes to the decrease in androgen level. The rs863108 polymorphism is a non-protein coding RNA in the LINC01524/LOC105372666 gene, which might play some roles in the pathogenesis of sPD through indirectly affecting the metabolism of androgen. The androgen hormone is closely associated with the pathogenesis of sPD, which is suggested to exert a protective effect in the brain (Snyder et al., 2018). Therefore, steroids and drugs altering endocrine conditions could have a potential curing effect for sPD. Sex hormones, particularly estrogen, progesterone, androgen, and dehydroepirosterone, play a protective effect in animal models and human studies of sPD. Drugs affecting the estrogen neurotransmission such as selective estrogen receptor modulators or affecting the steroid metabolism such as 5α-reductase inhibitors could be repositioned for the treatment of sPD. Sex steroids are also the modulator of neurotransmission; thus, they could repurpose to treat sPD motor symptoms and modulate the response to sPD medication (Bourque et al., 2019).

DMRT2, doublesex- and mab-3-related transcription factor 2, locates in 9p24.3, consists of nine exons, and is mainly expressed in the kidney, fat, lung, brain, colon, testis, lymph node, placenta, prostate, salivary gland, and esophagus (Fagerberg et al., 2014). DMRT2 shares a doublesex- and mab DNA-binding domain involved in sex determination. This gene also is associated with gonadal dysgenesis and XY sex reversal (Ottolenghi et al., 2000). Hence, this gene is one of candidates for sex-determining genes on chromosome 9.

SMARCA2 locates in 9p24.3 and consists of 38 exons. This gene comprehensively expresses in almost all tissues of the body and ubiquitously expresses in the ovary, testis, brain, thyroid, urinary bladder, and fat (Fagerberg et al., 2014). Possible functions consist of ATP binding, DNA-dependent ATPase activity (Xu et al., 2007), chromatin binding, helicase activity, histone binding, protein binding (Batsché et al., 2006; Tréand et al., 2006; Abramovitz et al., 2008; Kowenz-Leutz et al., 2010; Sahni et al., 2015; Garrido-Urbani et al., 2016), transcription coactivator activity (Abramovitz et al., 2008), and the DNA binding of transcription regulatory region (Abramovitz et al., 2008).

Our study showed that subjects carrying the T allele (T/T + G/T) of rs80315856 in the DMRT2/SMARCA2 gene increased the risk of sPD compared with other subjects. The relationship between DMRT2/SMARCA2 gene and sPD and their pathogeneses has not been studied yet. According to the above reviewed literatures, we suggested that the polymorphism of rs80315856 might be associated to the following elements. (1) The DMRT2 gene is a doublesex- and mab-3-related transcription factor, which decides the sexual gonadal genesis. Therefore, the alteration of rs80315856 polymorphism might affect the expression of male and female hormone, and the secretion of sexual hormone, especially the ratio of male and female hormone in a life time, might result in the imbalance of male and female hormone ratio following age increase, which results in sPD. The polymorphism alteration of DMRT2 gene might be one of the reasons of middle and elder population onset and the male domination of sPD. The polymorphism alteration of rs80315856 in the DMRT2/SMARCA2 gene was consistent with the rs863108 polymorphism in the LINC01524/LOC105372666 gene, which might further indicate that the disorder of sexual hormone secretion or metabolism was associated with the pathogenesis of sPD (Bourque et al., 2019). (2) The alteration of rs80315856 in the DMRT2/SMARCA2 gene might have an effect on DNA-binding transcription factor activity (Abramovitz et al., 2008), RNA polymerase II-specific, RNA polymerase II regulatory region sequence-specific DNA binding (Muchardt and Yaniv, 1993; Huuskonen et al., 2005; Xu et al., 2007), embryonic skeletal system development, the positive regulation of myotome development, the positive regulation of transcription by RNA polymerase II (Huuskonen et al., 2005; Xu et al., 2007), and the regulation of somitogenesis in the pathogensis of sPD. (3) The rs80315856 may also change the metal ion balance because of the change in metal ion binding and protein homodimerization activity, which might be associated with abnormal iron ion deposition in the pathogenesis of sPD (Jomova et al., 2010). (4) SMARCA2 gene codes ATP-dependent helicase that is associated with the actin-dependent regulator of chromatin a2, brahma homolog, global transcription activator homologous sequence, protein brahma homolog, and sucrose non-fermenting 2-like protein 2. Possible functions mainly take part in the metabolizing of energy in tissues and cells including the brain. The abnormal polymorphism of rs80315856 in the SMARCA2 gene might change the energy metabolization including the production of ATP and the balance of oxygen free radical generation and elimination. Our results further implied that the disorder of reactive oxygen species (ROS) is one of the possible pathogenesis of sPD, through regulating the transcription of certain genes by altering the chromatin structure around those genes, encoding the protein of partially large ATP-dependent chromatin, remodeling complex SNF/SWI, and generating the alternatively spliced transcript variants encoding different isoforms containing CAG length polymorphism.

PLEKHN1, also known as CLPABP, is pleckstrin homology domain containing N1. It locates in 1p36.33 and consists of 15 exons and is mainly expressed in the skin, esophagus, stomach, urinary bladder, and prostate (Fagerberg et al., 2014). PLEKHN1 majorly distributes in the cytoskeleton (Sano et al., 2008), mitochondrial membrane (Nishino et al., 2016; Kuriyama et al., 2018), mitochondrion (Sano et al., 2008; Maeda et al., 2018), and plasma membrane.

Our results showed that subjects carrying T allele (C/T + T/T) of rs28499371 in the PLEKHN1 gene decreased the risk of sPD compared with other subjects. The functional prediction of the mutation site and the structure domain analysis of rs28499371 revealed that missense mutation led to codon change from GCT to GTT and the amino acid change from A to V in the 43rd position of PLEKHN1 coding protein (Figure 3A). The pathogenicity prediction of the mutation site was benign, which might not result in pathogenicity. However, the structure domain analysis showed that the mutation sites located in the 143rd amino acid position and the main mutations are A, E, G, and S. In those mutations, A is the most important, and V is rare. The V mutation may lead to the pathogenicity (Figures 3D,E). The structure modeling analysis of rs28499371 found that the mutation amino acid is located at the center of the protein pocket, which may influence the neighborhood structure.

Besides our previous published study (Hu et al., 2016), the relationship and potential mechanism between PLEKHN1 and sPD have not been reported up to now. Because PLEKHN1 majorly distributes in the mitochondrion, we hypothesized that the alteration of rs28499371 polymorphism might be associated with the mitochondrion damage in the pathogenesis of sPD. Furthermore, respiratory chain impairment is a key feature in sPD patients, and there is a growing evidence that links proteins encoded by PD-associated genes to disturbances in the mitochondrial function (Grünewald et al., 2019). PLEKHN1 mainly takes part in the phospholipid-related functions including the phosphatidic acid, phosphatidylinositol phosphate, and phosphatidylserine binding (Sano et al., 2008); these functions play important biological effects in the production of nerve myelin sheath; moreover, PLEKHN1 also participates in the processes of 3′-UTR-mediated mRNA destabilization (Maeda et al., 2018), positive apoptotic regulation, and hypoxia response (Kuriyama et al., 2018). Therefore, we suggest that the alteration of rs28499371 polymorphism in the PLEKHN1 gene might play some pathophysiological roles in the pathogenesis of sPD through affecting the production of nerve myelin sheath, the destabilization of 3′-UTR-mediated mRNA, the regulation of neural cell apoptosis, and the response of hypoxia, subsequently contributing to nerve myelin sheath deletion, excessive neural cell apoptosis, and increased ROS production, which ultimately result in the death of the DA neuron in sPD.

FLJ23172, also known as TMEM212 gene, locates in 3q26.31, consists of five exons, and majorly expresses in the lung, endometrium, testis, and prostate (Fagerberg et al., 2014). FNDC3B, also known as FAD104, PRO4979, and YVTM2421, locates in 3q26.31, consists of 30 exons, and ubiquitously expresses in the ovary, testis, brain, thyroid, urinary bladder, fat, adrenal tissue, and endometrium (Fagerberg et al., 2014). This gene codes the protein of fibronectin type III domain-containing protein 3B and also includes HCV NS5A-binding protein 37 and factor for adipocyte differentiation 104. The possible function is RNA binding (Castello et al., 2012).

Our results revealed that subjects carrying the T allele (T/T + C/T) of rs2270568 exhibited no significance when compared with other subjects, and the G/T genotype of rs2270568 decreased the risk of sPD when compared with other genotypes (Table 3). The MAF of rs2270568 was significantly higher in sPD patients than that in controls, which implied that the T allele of rs2270568 was a risk locus for sPD. The pathogenicity prediction and the structure modeling of rs2270568 indicated that the mutation site of rs2270568 resulted in a nucleotide alteration from T to C (Figure 5A). The variant is synonymous and resides within a pathogenicity region, which may suggest its potential involvement in the disease (Figure 5B). Although the mutation site appeared between the coil and beta sheet, which were the pathogenicity mutation region with high frequency occurrence, this protein function might not be influenced by the mutation site. However, the pathogenicity sites near the mutation site might influence the protein function and the disease regulation mechanism (Figure 5C). FLJ23172 majorly expresses in the testis and prostate; therefore, we speculated that alteration of rs2270568 polymorphism might affect the secretion of androgen and progesterone and participate in the pathogenesis of sPD (Bourque et al., 2019). FNDC3B ubiquitously expresses in the testis, brain, and adrenal tissues, which implied that the rs2270568 polymorphism alteration might regulate the secretion of prostaglandin, androgen, and adrenocortical hormones, and play some effects in sPD (Bourque et al., 2019). The accurate mechanisms about the relationship between FLJ23172/FNDC3B and the pathogenesis of sPD need to be further studied.

## Conclusion

**FIGURE 6:**
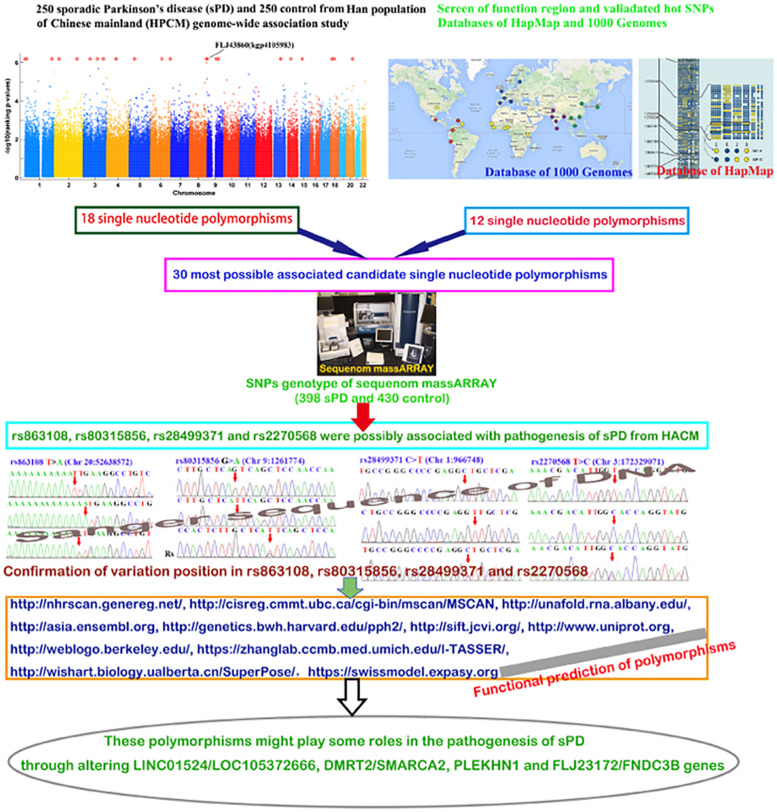
The studied diagrammatic sketch. This figure generally described the design, procedures, methods, results, and conclusion.

In general, our data supported that four polymorphisms of rs863108, rs80315856, rs28499371, and rs2270568 possibly were associated with the pathogenesis of sPD in HACM and further identified two previous reported SNPs of rs863108 and rs80315856. The alteration of rs863108, rs80315856, rs28499371, and rs2270568 polymorphism might affect the metabolism of sexual hormones including estrogen, progesterone, androgen, dehydroepirosterone, prostaglandin, and adrenocortical hormones; the increase in ROS production induced by abnormal iron ion deposition and hypoxia response; the increase in neural cell apoptosis; and the deletion of nerve myelin sheath in the pathogenesis of sPD in HACM (Figure 6).

## Data Availability Statement

The original contributions presented in the study are included in the article/Supplementary Material, further inquiries can be directed to the corresponding authors.

## Ethics Statement

This study was approved by the Institutional Review Board of the Hospital Human Ethics Committee of The First Affiliated Hospital of Nanchang University and The Affiliated Guangdong General Hospital of Nan Fang Medical University. The patients/participants provided their written informed consent to participate in this study.

## Author Contributions

RX, BW, and XZ conceived and designed the experiments and wrote the manuscript. BW, XL, SX, ZL, and YZ performed the experiments and analyzed the data. RX contributed to the reagents, materials, and analysis tools. RX and BW processed the figures. All authors have been involved in the drafting, critical revision, and final approval of the manuscript for publication, and agreed to be accountable for all aspects of the work in ensuring that questions related to the accuracy or integrity of any part of the work are appropriately investigated and resolved.

## Conflict of Interest

The authors declare that the research was conducted in the absence of any commercial or financial relationships that could be construed as a potential conflict of interest.

## Footnotes

1 http://hapmap.ncbi.nlm.nih.gov/

2 http://browser.1000genomes.org/

3 http://cisreg.cmmt.ubc.ca/cgi-bin/mscan/MSCAN

4 http://genetics.bwh.harvard.edu/pph2/

5 http://sift.jcvi.org/

6 http://www.uniprot.org

7 http://weblogo.berkeley.edu/

8 https://zhanglab.ccmb.med.umich.edu/I-TASSER/

9 http://wishart.biology.ualberta.cn/SuperPose/

10 https://swissmodel.expasy.org

11 https://www.ncbi.nlm.nih.gov/snp/

12 http://weblogo.berkeley.edu/

## References

[B1] Abramovitz L. Shapira T. Ben-Dror I. Dror V. Granot L. Rousso T. (2008). Dual role of NRSF/REST in activation and repression of the glucocorticoid response. *J. Biol. Chem.* 283 110–119. 10.1074/jbc.M707366200 17984088

[B2] Alkema W. B. Johansson O. Lagergren J. Wasserman W. W. (2004). MSCAN: identification of functional clusters of transcription factor binding sites. *Nucleic. Acids. Res.* 32 W195–W198. 10.1093/nar/gkh387 15215379 PMC441525

[B3] Allen H. L. Estrada K. Lettre G. Berndt S. I. Weedon M. N. Rivadeneira F. (2010). Hundreds of variants clustered in genomic loci and biological pathways affect human height. *Nature* 467 832–838. 10.1038/nature09410 20881960 PMC2955183

[B4] Batsché E. Yaniv M. Muchardt C. (2006). The human SWI/SNF subunit Brm is a regulator of alternative splicing. *Nat. Struct. Mol Biol.* 13 22–29. 10.1038/nsmb1030 16341228

[B5] Billingsley K. J. Bandres-Ciga S. Saez-Atienzar S. Singleton A. B. (2018). Genetic risk factors in Parkinson’s disease. *Cell Tissue Res.* 373 9–20. 29536161 10.1007/s00441-018-2817-y PMC6201690

[B6] Bourque M. Morissette M. Di Paolo T. (2019). Repurposing sex steroids and related drugs as potential treatment for Parkinson’s disease. *Neuropharmacology* 147 37–54. 10.1016/j.neuropharm.2018.04.005 29649433

[B7] Castello A. Fischer B. Eichelbaum K. Horos R. Beckmann B. M. Strein C. (2012). Insights into RNA biology from an atlas of mammalian mRNA-binding proteins. *Cell* 149 1393–1406. 10.1016/j.cell.2012.04.031 22658674

[B8] Cummings J. L. Henchcliffe C. Schaier S. Simuni T. Waxman A. Kemp P. (2011). The role of dopaminergic imaging in patients with symptoms of dopaminergic system neurodegeneration. *Brain* 134 3146–3166. 10.1093/brain/awr177 21810889

[B9] Fagerberg L. Hallström B. M. Oksvold P. Kampf C. Djureinovic D. Odeberg J. (2014). Analysis of the human tissue-specific expression by genome-wide integration of transcriptomics and antibody-based proteomics. *Mol. Cell Proteomics.* 13 397–406. 10.1074/mcp.M113.035600 24309898 PMC3916642

[B10] Fahn S. (2003). Description of Parkinson’s disease as a clinical syndrome. *Ann. N. Y. Acad. Sci.* 991 1–14. 10.1111/j.1749-6632.2003.tb07458.x 12846969

[B11] Garrido-Urbani S. Garg P. Ghossoub R. Arnold R. Lembo F. Sundell G. N. (2016). Proteomic peptide phage display uncovers novel interactions of the PDZ1-2 supramodule of syntenin. *FEBS. Lett.* 590 3–12. 10.1002/1873-3468.12037 26787460 PMC4819696

[B12] Göttlich M. Münte T. F. Heldmann M. Kasten M. Hagenah J. Krämer U. M. (2013). Altered resting state brain networks in Parkinson’s disease. *PLoS. One* 8:e77336. 10.1371/journal.pone.0077336 24204812 PMC3810472

[B13] Grünewald A. Kumar K. R. Sue C. M. (2019). New insights into the complex role of mitochondria in Parkinson’s disease. *Prog. Neurobiol.* 177 73–93. 10.1016/j.pneurobio.2018.09.003 30219247

[B14] Hellquist A. Zucchelli M. Lindgren C. M. Saarialho-Kere U. Järvinen T. M. Koskenmies S. (2009). Identification of MAMDC1 as a candidate susceptibility gene for systemic lupus erythematosus (SLE). *PLoS One* 4:e8037. 10.1371/journal.pone.0008037 19997561 PMC2785483

[B15] Huuskonen J. Vishnu M. Fielding P. E. Fielding C. J. (2005). Activation of ATP-binding cassette transporter A1 transcription by chromatin remodeling complex. *Arterioscler. Thromb. Vasc. Biol.* 25 1180–1185. 10.1161/01.ATV.0000163186.58462.c5 15774904

[B16] Hu Y. Deng L. Zhang J. Fang X. Mei P. Cao X. (2016). A pooling genome-wide association study combining a pathway analysis for typical sporadic Parkinson’s disease in the han population of Chinese Mainland. *Mol. Neurobiol.* 53 4302–4318. 10.1007/s12035-015-9331-y 26227905

[B17] Jomova K. Vondrakova D. Lawson M. Valko M. (2010). Metals, oxidative stress and neurodegenerative disorders. *Mol. Cell Biochem.* 345 91–104. 10.1007/s11010-010-0563-x 20730621

[B18] Kalia L. V. Lang A. E. (2015). Parkinson’s disease. *Lancet* 386 896–912. 10.1016/S0140-6736(14)61393-3 25904081

[B19] Khoo T. K. Yarnall A. J. Duncan G. W. Coleman S. O’Brien J. T. Brooks D. J. (2013). The spectrum of nonmotor symptoms in early Parkinson disease. *Neurology* 80 276–281. 10.1212/WNL.0b013e31827deb74 23319473 PMC3589180

[B20] Kowenz-Leutz E. Pless O. Dittmar G. Knoblich M. Leutz A. (2010). Crosstalk between C/EBPbeta phosphorylation, arginine methylation, and SWI/SNF/Mediator implies an indexing transcription factor code. *EMBO. J.* 29 1105–1115. 10.1038/emboj.2010.3 20111005 PMC2845275

[B21] Kuriyama S. Tsuji T. Sakuma T. (2018). PLEKHN1 promotes apoptosis by enhancing Bax-Bak hetro-oligomerization through interaction with Bid in human colon cancer. *Cell Death Discov.* 4:11. 10.1038/s41420-017-0006-5 29531808 PMC5841295

[B22] Li J. Jin M. Wang L. Qin B. Wang K. (2017). MDS clinical diagnostic criteria for Parkinson’s disease in China. *J. Neurol.* 264 476–481. 10.1007/s00415-016-8370-2 28025665

[B23] Li S. Cui Y. Romero R. (2014). Entropy-based selection for maternal-fetal genotype incompatibility with application to preterm prelabor rupture of membranes. *BMC Genet.* 15:66. 10.1186/1471-2156-15-66 24916189 PMC4057811

[B24] Maeda A. Uchida M. Nishikawa S. Nishino T. Konishi H. (2018). Role of N-myristoylation in stability and subcellular localization of the CLPABP protein. *Biochem. Biophys. Res. Commun.* 495 1249–1256. 10.1016/j.bbrc.2017.11.112 29180010

[B25] Marsden C. D. (1994). Parkinson’s disease. *J. Neurol. Neurosurg. Psychiatry* 57 672–681. 10.1136/jnnp.57.6.672 7755681 PMC1072968

[B26] Muchardt C. Yaniv M. (1993). A human homologue of Saccharomyces cerevisiae SNF2/SWI2 and Drosophila brm genes potentiates transcriptional activation by the glucocorticoid receptor. *EMBO. J.* 12 4279–4290. 10.1002/j.1460-2075.1993.tb06112.x 8223438 PMC413724

[B27] Nishino T. Matsunaga R. Jikihara H. Uchida M. Maeda A. Qi G. (2016). Antagonizing effect of CLPABP on the function of HuR as a regulator of ARE-containing leptin mRNA stability and the effect of its depletion on obesity in old male mouse. *Biochim. Biophys. Acta* 1861 1816–1827. 10.1016/j.bbalip.2016.09.006 27616329

[B28] Ottolenghi C. Veitia R. Barbieri M. Fellous M. McElreavey K. (2000). The human doublesex-related gene, DMRT2, is homologous to a gene involved in somitogenesis and encodes a potential bicistronic transcript. *Genomics* 64 179–186. 10.1006/geno.2000.6120 10729224

[B29] Polito L. Greco A. Seripa D. (2016). Genetic profile, environmental exposure, and their interaction in Parkinson’s Disease. *Parkinsons Dis.* 2016:6465793. 10.1155/2016/6465793 26942037 PMC4752982

[B30] Rozenkrantz L. Gan-Or Z. Gana-Weisz M. Mirelman A. Giladi N. Bar-Shira A. (2016). SEPT14 Is associated with a reduced risk for Parkinson’s disease and expressed in human brain. *J. Mol. Neurosci.* 59 343–350. 10.1007/s12031-016-0738-3 27115672

[B31] Sahni N. Yi S. Taipale M. Bass J. I. J. Coulombe-Huntington J. Yang F. (2015). Widespread macromolecular interaction perturbations in human genetic disorders. *Cell* 161 647–660. 10.1016/j.cell.2015.04.013 25910212 PMC4441215

[B32] Sano E. Shono S. Tashiro K. Konishi H. Yamauchi E. Taniguchi H. (2008). Novel tyrosine phosphorylated and cardiolipin- binding protein CLPABP functions as mitochondrial RNA granule. *Biochim. Biophys. Acta* 1783 1036–1047. 10.1016/j.bbamcr.2007 18191643

[B33] Singleton A. B. Farrer M. J. Bonifati V. (2013). The genetics of Parkinson’s disease: progress and therapeutic implications. *Mov. Disord.* 28 14–23. 10.1002/mds.25249 23389780 PMC3578399

[B34] Snyder B. Duong P. Trieu J. Cunningham R. L. (2018). Androgens modulate chronic intermittent hypoxia effects on brain and behavior. *Horm. Behav.* 106 62–73. 10.1016/j.yhbeh.2018.09.005 30268884 PMC6486829

[B35] Soldner F. Stelzer Y. Shivalila C. S. Abraham B. J. Latourelle J. C. Barrasa M. I. (2016). Parkinson-associated risk variant in distal enhancer of alpha-synuclein modulates target gene expression. *Nature* 533 95–99. 10.1038/nature17939 27096366 PMC5042324

[B36] Strausberg R. L. Feingold E. A. Grouse L. H. Derge J. G. Klausner R. D. Collins F. S. (2002). Generation and initial analysis of more than 15,000 full-length human and mouse cDNA sequences. *Proc. Natl. Acad. Sci. USA.* 99 16899–16903. 10.1073/pnas.242603899 12477932 PMC139241

[B37] Tréand C. du, Chéné I. Brès V. Kiernan R. Benarous R. (2006). Requirement for SWI/SNF chromatin-remodeling complex in Tat-mediated activation of the HIV-1 promoter. *EMBO J.* 25 1690–1699. 10.1038/sj.emboj.7601074 16601680 PMC1440843

[B38] von Otter M. Bergström P. Quattrone A. De Marco E. V. Annesi G. Söderkvist P. (2014). Genetic associations of Nrf2-encoding NFE2L2 variants with Parkinson’s disease-a multicenter study. *BMC Med. Genet.* 15:131. 10.1186/s12881-014-0131-4 25496089 PMC4335439

[B39] Wang L. Maldonado L. Beecham G. W. Martin E. R. Evatt M. L. Ritchie J. C. (2016). DNA variants in CACNA1C modify Parkinson disease risk only when vitamin D level is deficient. *Neurol. Genet.* 2:e72. 10.1212/NXG.0000000000000072 27123490 PMC4830205

[B40] Xu Y. Zhang J. Chen X. (2007). The activity of p53 is differentially regulated by Brm- and Brg1-containing SWI/SNF chromatin remodeling complexes. *J. Biol. Chem.* 282 37429–37435. 10.1074/jbc.M706039200 17938176

[B41] Yang X. Zhao Q. An R. Zhou H. Lin Z. Xu Y. (2016). SNP rs1805874 of the calbindin1 gene is associated with Parkinson’s disease in Han Chinese. *Genet. Test. Mol. Biomarkers* 20 753–757. 10.1089/gtmb.2016.0149 27611799

