# Supplementary material for: Sporadic Parkinson’s Disease Potential Risk Loci Identified in Han Ancestry of Chinese Mainland
Source: Front Aging Neurosci. 2021 Jan 12;12:603793. doi: 10.3389/fnagi.2020.603793 (PMC7835639; doi:10.3389/fnagi.2020.603793)
Supplement: Supplementary file 1 [file Data_Sheet_1.PDF]

Supplementary Table 1 Possible susceptible SNPs of sPD based on the result of function region SNPs and valiadated hot SNPs screen

Functional regional SNPs (CNTNAP2)							
rs number	Chr	MAF in (CHB/HCB)	Location	Function	Function Prediction	Source	Reference
rs10240503	7	0.189	147977886	cds-synon	-	1000 Genomes	CNTNAP2 variants affect early language development in the general population、Free the data: one laboratory's approach to knowledge-based genomic variant classification and preparation for EMR integration of genomic data.
rs1062072	7	0.383	148417773	3'UTR	-	1000 Genomes	No reference
rs2462603	7	0.311	146116762	5'UTR	TFBS	1000 Genomes	No reference
rs2530310	7	0.34	148420550	3'UTR	miRNA(miRanda)	1000 Genomes	miR-485-5p binding site SNP rs8752 in HPGD gene is associated with breast cancer risk.
rs2530311	7	0.383	148419358	3'UTR	miRNA(miRanda)	1000 Genomes	No reference
rs2530312	7	0.388	148416803	3'UTR	miRNA(miRanda)	1000 Genomes	No reference
rs2717829	7	0.257	148420413	3'UTR	miRNA(miRanda)	1000 Genomes	No reference
rs3194	7	0.325	148417173	3'UTR	miRNA(miRanda)	1000 Genomes	No reference
rs9648691	7	0.383	148409398	cds-synon	Splicing(ESE or ESS)	1000 Genomes	Free the data: one laboratory's approach to knowledge-based genomic variant classification and preparation for EMR integration of genomic data.
rs987456	7	0.301	148415895	3'UTR	miRNA(miRanda)	1000 Genomes	No reference

Continued									
Validated Hot SNPs (CNTNAP2)									
rs number	Chr	MAF in (CHB/HCB)	Location	Function	Function Prediction	Source	Reference	Comment	Disease
rs10244837	7	0.485	146778222	intron	-	1000 Genomes	Association analysis of CNTNAP2 polymorphisms with autism in the Chinese Han population.	The results show that a common	autism

								noncoding variant (rs10500171) is associated with the increased risk for autism, and haplotype T-A (rs7794745-rs10500171, P=0.011) and haplotype A-T-A (rs10244837-rs7794745-rs10500171, P=0.032) also showed evidence of association.	
rs10500171	7	0.388	147183313	intron	-	1000 Genomes	Association analysis of CNTNAP2 polymorphisms with autism in the Chinese Han population.	The results show that a common noncoding variant (rs10500171) is associated with the increased	autism

								risk for autism, and haplotype T-A (rs7794745-rs10500171, P=0.011) and haplotype A-T-A (rs10244837-rs7794745-rs10500171, P=0.032) also showed evidence of association.	
rs1404699	7	0.451	147343214	intron	-	1000 Genomes	Evaluation of CNTNAP2 gene polymorphisms for exfoliation syndrome in Japanese.	The allele frequencies of rs1404699 (p=8.57XE-3, odds ratio (OR)=1.59, 95% confidential intervals (CI); 1,12–2.24) and rs7803992 (p=5.43XE-4, OR=1.86, 95%	exfoliation syndrome

								CI; 1.31–2.65) were statistically significantly different between XFS and controls.	
rs2107856	7	0.388	147491593	intron	-	1000 Genomes	Evaluation of CNTNAP2 gene polymorphisms for exfoliation syndrome in Japanese.	The allele and the genotype frequencies of rs2107856 and rs2141388, which were statistically significant SNPs in an earlier study.	exfoliation syndrome
rs2141388	7	0.388	147492648	intron	-	1000 Genomes	Evaluation of CNTNAP2 gene polymorphisms for exfoliation syndrome in Japanese.	The allele and the genotype frequencies of rs2107856 and rs2141388, which were statistically significant SNPs in an earlier study.	exfoliation syndrome
rs2215798	7	0.165	147889489	intron	-	1000 Genomes	Defining the Contribution of CNTNAP2 to Autism Susceptibility.	(rs17170073, p = 2.0 x 10-4; rs2215798, p = 1.6 x 10-4)	autism
rs2253031	7	0.248	147934809	intron	-	1000 Genomes	Defining the Contribution of CNTNAP2 to	two highly correlated	autism

							Autism Susceptibility.	(r2 = 0.99) SNPs in intron 14 showed significant association with autism (rs2710093, p = 9.0 x 10-6; rs2253031, p = 2.5 x 10-5).	
rs2538991	7	0.422	147882527	intron	-	1000 Genomes	CNTNAP2 polymorphisms and structural brain connectivity: A diffusion-tensor imaging study.	associated with anterior-posterior functional connectivity	structural brain connectivity
rs2710093	7	0.248	147935043	intron	-	1000 Genomes	Defining the Contribution of CNTNAP2 to Autism Susceptibility.	two highly correlated (r2 = 0.99) SNPs in intron 14	autism
								showed significant association with autism (rs2710093, p = 9.0 x 10-6; rs2253031, p = 2.5 x 10-5)	

rs2710102	7	0.422	147877298	intron	-	1000 Genomes	1. CNTNAP2 polymorphisms and structural brain connectivity: A diffusion-tensor imaging study.	associated with anterior-posterior functional connectivity	structural brain connectivity
							2. A Common Genetic Variant in the Neurexin Superfamily Member CNTNAP2 Is Associated with Increased Risk for Selective Mutism and Social Anxiety-Related Traits.	Analyses revealed nominal significance (p = .018) for association of SM with rs2710102, which, with rs6944808, was part of a common haplotype associated with SM (permutation p = .022). Adjusting for sex and ancestral proportion, each copy of the rs2710102*a risk allele in the young adults was associated with increased odds of being >1 SD above the mean on the Social Interactiona	Selective Mutism

								l Anxiety Scale (odds ratio = 1.33, p = .015) and Retrospective Self-Report of Inhibition (odds ratio = 1.40, p = .010).	
rs6944808	7	0.379	147555456	intron	-	1000 Genomes	A Common Genetic Variant in the Neurexin Superfamily Member CNTNAP2 Is Associated with Increased Risk for Selective Mutism and Social Anxiety-Related Traits.		Selective Mutism
rs759178	7	0.422	147878020	intron	-	1000 Genomes	CNTNAP2 polymorphisms and structural brain connectivity: A diffusion-tensor imaging study.	associated with anterior-posterior functional connectivity	structural brain connectivity
rs7794745	7	0.442	146792514	intron	-	1000 Genomes	1. Normal variation in fronto-occipital circuitry and cerebellar structure with an autism-associated polymorphism of CNTNAP2.	Homozygotes for the risk allele showed significant reductions in grey and white matter volume and fractional anisotropy in several regions that have already been implicated in ASD, including the cerebellum, fusiform gyrus, occipital and	autism

								frontal cortices.	
							2. Association analysis of CNTNAP2 polymorphisms with autism in the Chinese Han population.	The results show that a common noncoding variant (rs10500171) is associated with the increased risk for autism, and haplotype T-A (rs7794745-rs10500171, P=0.011) and haplotype A-T-A (rs10244837-rs7794745-rs10500171, P=0.032) also showed evidence of association.	autism
rs7803992	7	0.451	147343905	intron	-	1000 Genomes	Evaluation of CNTNAP2 gene polymorphisms for exfoliation syndrome in Japanese.	The allele frequencies of rs1404699 (p=8.57XE-3, odds ratio (OR)=1.59, 95% confidential intervals (CI); 1,12–2.24) and rs7803992	exfoliation syndrome

								(p=5.43XE-4, OR=1.86, 95% CI; 1.31–2.65) were statistically significantly different between XFS and controls.	

Supplementary Table 1 (Continued)

Functional regional SNPs (CSMD1)							
rs number	Chr	MAF in (CHB/HCB)	Location	Function	Function Prediction	Source	Reference
rs10088378	8	0.398	3408068	cds-synon	nsSNP	1000 Genomes	No association
rs1111656	8	0.083	4528292	5‘UTR	-	1000 Genomes	No references
rs111941841	8	0.286	3021762	cds-synon	-	1000 Genomes	No references
rs117824961	8	0.136	4531872	cds-synon	-	1000 Genomes	No references
rs13249778	8	0.413	4532125	missense	-	1000 Genomes	No references
rs13252998	8	0.364	4531965	cds-synon	-	1000 Genomes	No references
rs13260153	8	0.413	3022586	3‘UTR	-	1000 Genomes	No references
rs17066296	8	0.155	3493625	cds-synon	-	1000 Genomes	No association
rs17070498	8	0.194	4533515	3‘UTR	-	1000 Genomes	No references
rs17079099	8	0.388	3021548	missense	-	1000 Genomes	No references
rs17079101	8	0.097	3023660	3‘UTR	-	1000 Genomes	No references
rs17317488	8	0.097	3023059	3‘UTR	-	1000 Genomes	No references
rs2161752	8	0.087	3343355	cds-synon	Splicing(ESE or ESS)、nsSNP	1000 Genomes	No association

rs2291319	8	0.306	2937908	3‘UTR	-	1000 Genomes	No references
rs28455997	8	0.063	3219437	missense	Splicing(ESE or ESS)	1000 Genomes	Altered CSMD1 Expression Alters Cocaine-Conditioned Place Preference: Mutual Support for a Complex Locus from Human and Mouse Models.
rs35043129	8	0.136	2950261	cds-synon	Splicing(ESE or ESS)、Splicing(abolish domain)、Stop Codon	1000 Genomes	No association
rs3802303	8	0.073	3396246	cds-synon	Splicing(ESE or ESS)、nsSNP	1000 Genomes	No association
rs4875703	8	0.359	3367039	cds-synon	Splicing(ESE or ESS)、nsSNP	1000 Genomes	No association
rs4876056	8	0.092	2962521	cds-synon	Splicing(ESE or ESS)、Splicing(abolish domain)、nsSNP	1000 Genomes	No association
rs55958324	8	0.083	2935559	3‘UTR	miRNA(miRanda)	1000 Genomes	No references
rs56109797	8	0.136	3493673	cds-synon	-	1000 Genomes	No references
rs59265291	8	0.5	4533007	3‘UTR	-	1000 Genomes	No references
rs592700	8	0.107	3008823	5‘UTR	-	1000 Genomes	No references
rs60392405	8	0.359	4533022	3‘UTR	-	1000 Genomes	No references
rs607559	8	0.388	3022166	missense	-	1000 Genomes	No references
rs62487758	8	0.189	3022260	cds-synon	-	1000 Genomes	No references
rs6558702	8	0.136	3052470	missense	Splicing(ESE or ESS)	1000 Genomes	Altered CSMD1 Expression Alters Cocaine-Conditioned Place Preference: Mutual Support for a Complex Locus from Human and Mouse Models.
rs667595	8	0.146	2974617	cds-synon	Splicing(ESE or	1000 Genomes	No references

					ESS)、nsSNP		
rs667859	8	0.422	2963223	cds-synon	nsSNP	1000 Genomes	No references
rs673430	8	0.087	2935780	3‘UTR	miRNA(miRanda)	1000 Genomes	No references
rs73183587	8	0.049	3214585	cds-synon	-	1000 Genomes	No references
rs74495891	8	0.277	3022999	3‘UTR	-	1000 Genomes	No references
rs74612718	8	0.286	3021948	cds-synon	-	1000 Genomes	No references
rs75505851	8	0.16	2936551	3‘UTR	-	1000 Genomes	No references
rs77085491	8	0.286	3021883	missense	-	1000 Genomes	No references
rs7824683	8	0.087	3024198	3‘UTR	-	1000 Genomes	No references

Continued

Validated Hot SNPs (CSMD1)									
rs number	Chr	MAF in (CHB/HCB)	Location	Function	Function Prediction	Source	Reference	Comment	Disease
rs1529316	8	0.16	3970616	intron	-	1000 Genomes	Replication of top markers of a genome-wide association study in multiple sclerosis in Spain.	Two polymorphisms in the CSMD1 gene were among the top associated markers originally found in the GWAS by Baranzini et al.14: rs1529316 and rs2049306 (r2=0.84).	multiple sclerosis
rs1611927	8	0.34	4741721	intron	-	1000 Genomes	Replication of top markers of a genome-wide association study in multiple sclerosis in Spain.	Another polymorphism in this gene, rs1611927, was recently found associated in a meta-analysis.	multiple sclerosis

rs2049306	8	0.209	3974558	intron	-	1000 Genomes	Replication of top markers of a genome-wide association study in multiple sclerosis in Spain.	Two polymorphisms in the CSMD1 gene were among the top associated markers originally found in the GWAS by Baranzini et al.14: rs1529316 and rs2049306 (r2=0.84).	multiple sclerosis
rs2554503	8	0.117	3967303	intron	-	1000 Genomes	Identification of Evidence Suggestive of an Association with Peripheral Arterial Disease at the OSBPL10 Locus by Genome-Wide Investigation in the Japanese Population.	PAD was modestly associated at several other loci such as rs2554503 in CSMD1 (p=5.7E-5; OR=1.32, 95% CI 1.15-1.51)	Peripheral arterial disease (PAD)
rs7007032	8	0.214	3821924	intron	-	1000 Genomes	Study on association of rs7007032 polymorphism within CSMD1 gene with some	significant differences between clinical subgroups and controls(P=1.0×10-4)	psoriasis vulgaris

							clinical phenotypes of psoriasis vulgaris in Chinese Han population.		

Supplementary Table 1 (Continued)

Functional regional SNPs (DAB1)							
rs number	Chr	MAF in (CHB/HCB)	Location	Function	Function Prediction	Source	Reference
rs79902981	1	0.053	58250464	5‘UTR	-	1000 Genomes	No references

Continued							
Validated Hot SNPs (DAB1)							
rs number	MAF in (CHB/HCB)	Function	Function Prediction	Source	Reference	Comment	Disease
rs1202773	0.165	intron	-	1000 Genomes	Association study between genes in Reelin signaling pathway and autism identifies DAB1 as a susceptibility gene in a Chinese Han population.	we found significant genetic association between autism and four SNPs in DAB1 (rs12035887 G: p = 0.0006; rs3738556 G: p = 0.0044; rs1202773 A: p = 0.0048; rs12740765 T: p = 0.0196).	autism

rs12035887	0.354	intron	-	1000 Genomes	Association study between genes in Reelin signaling pathway and autism identifies DAB1 as a susceptibility gene in a Chinese Han population.	we found significant genetic association between autism and four SNPs in DAB1 (rs12035887 G: p = 0.0006; rs3738556 G: p = 0.0044; rs1202773 A: p = 0.0048; rs12740765 T: p =0.0196).	autism
rs12740765	0.199	intron	-	1000 Genomes	Association study between genes in Reelin signaling pathway and autism identifies DAB1 as a susceptibility gene in a Chinese Han population.	we found significant genetic association between autism and four SNPs in DAB1 (rs12035887 G: p = 0.0006; rs3738556 G: p = 0.0044; rs1202773 A: p = 0.0048; rs12740765 T: p = 0.0196).	autism
rs3738556	0.49	intron	-	1000 Genomes	Association study between genes in Reelin signaling pathway and autism identifies DAB1 as a susceptibility gene in a Chinese Han population.	we found significant genetic association between autism and four SNPs in DAB1 (rs12035887 G: p = 0.0006; rs3738556 G: p = 0.0044; rs1202773 A: p = 0.0048; rs12740765 T: p = 0.0196).	autism

Supplementary Table 1 (Continued)

Functional regional SNPs (DPP6)							
rs number	MAF in (CHB/HCB)	Chr	Location	Function	Function Prediction	Source	Reference
rs10226961	0.447	7	154063845	missense	-	1000 Genomes	No references
rs10240633	0.422	7	154063836	cds-synon	-	1000 Genomes	No references
rs1047053	0.282	7	154893261	3‘UTR	miRNA(miRanda)	1000 Genomes	No association

rs1047064	0.16	7	154894095	3‘UTR	miRNA(miRanda)	1000 Genomes	No association
rs11243339	0.092	7	154637850	cds-synon	Splicing(ESE or ESS)	1000 Genomes	No association
rs1129300	0.447	7	154889340	cds-synon	Splicing(ESE or ESS)	1000 Genomes	No references
rs1129301	0.447	7	154889506	cds-synon	Splicing(ESE or ESS)	1000 Genomes	No references
rs12670419	0.456	7	154054590	3‘UTR	-	1000 Genomes	No references
rs12674376	0.17	7	154893511	3‘UTR	miRNA(miRanda)	1000 Genomes	No references
rs138291928	0.49	7	154058678	missense	-	1000 Genomes	No references
rs140470029	0.112	7	154061058	cds-synon	-	1000 Genomes	No references
rs140597091	0.272	7	154060558	missense	-	1000 Genomes	No references
rs142406463	0.058	7	154064735	5‘UTR	-	1000 Genomes	No references
rs146739046	0.233	7	154,893,200 - 154,893,201	3‘UTR	-	1000 Genomes	No references
rs1525752	0.296	7	154893902	3‘UTR	miRNA(miRanda) 、 miRNA(Sanger)	1000 Genomes	No references
rs1860503	0.354	7	154055470	cds-synon, 3‘UTR	-	1000 Genomes	No references
rs1974615	0.451	7	154052668	5‘UTR	TFBS	1000 Genomes	No references
rs2293353	0.49	7	154875918	cds-synon	Splicing(ESE or ESS)	1000 Genomes	No association
rs2429610	0.359	7	154057537	cds-synon	-	1000 Genomes	No references
rs2533722	0.354	7	154055354	3‘UTR	-	1000 Genomes	No references
rs2533726	0.354	7	154054814	3‘UTR	-	1000 Genomes	No references
rs2628674	0.359	7	154055217	3‘UTR	-	1000 Genomes	No references
rs28706744	0.063	7	154053562	missense	-	1000 Genomes	No references
rs3179887	0.354	7	154055902	cds-synon，3‘UTR，missense	-	1000 Genomes	No references
rs3298	0.063	7	154894163	3‘UTR	miRNA(miRanda)	1000 Genomes	No references

rs3734960	0.248	7	154892443	missense, cds-synon	nsSNP	1000 Genomes	No references
rs3734961	0.17	7	154893852	3‘UTR	miRNA(miRanda)	1000 Genomes	No references
rs374262699	0.485	7	154,052,688 - 154,052,690	5‘UTR	-	1000 Genomes	No references
rs3807218	0.16	7	154669402	cds-synon	Splicing(ESE or ESS) 、Splicing(abolish domain)	1000 Genomes	Haplotype-sharing analysis implicates chromosome 7q36 harboring DPP6 in familial idiopathic ventricular fibrillation.
rs3817522	0.121	7	154880936	cds-synon	Splicing(ESE or ESS) 、Splicing(abolish domain)	1000 Genomes	No association
rs4067507	0.481	7	154065569	5‘UTR	-	1000 Genomes	No references
rs4067508	0.481	7	154065563	5‘UTR	-	1000 Genomes	No references
rs4725520	0.131	7	154054570	3‘UTR	-	1000 Genomes	No references
rs56091483	0.092	7	154769478	cds-synon	Splicing(ESE or ESS)	1000 Genomes	No association
rs56404363	0.364	7	154059462	missense	-	1000 Genomes	No references
rs571183490	0.5	7	154052721	5‘UTR	-	1000 Genomes	No references
rs6464382	0.49	7	154064114	5‘UTR	-	1000 Genomes	No references
rs77985994	0.083	7	154588278	3‘UTR	-	1000 Genomes	No references
rs79536510	0.126	7	154055705	missense, 3‘UTR	-	1000 Genomes	No references

Continued

Validated Hot SNPs (DPP6)

rs number	MAF in (CHB/HCB)	Chr	Location	Function	Function Prediction	Source	Reference	Comment	Disease
rs10260404	0.165	7	154513713	intron	-	1000 Genomes	DPP6 gene variability confers		Amyotrophic

							increased risk of developing sporadic amyotrophic lateral sclerosis in Italian patients.		lateral sclerosis
rs11767658	0.248	7	154640535	intron	-	1000 Genomes	Association between DPP6 polymorphism and the risk of progressive multiple sclerosis in Northern and Southern Europeans.	This gene emerged as a candidate gene in a genome-wide association study (GWAS) performed in an Italian sample of PrMS and controls in which two SNPs located in the gene (rs6956703 and rs11767658) showed evidence of association (nominal p-value < 10−4).	progressive forms of multiple sclerosis (PrMS)
rs2046748	0.466	7	154622300	intron	-	1000 Genomes	Association between DPP6 polymorphism and the risk of progressive multiple sclerosis in	combined analysis confirmed the	progressive forms of multiple

							Northern and Southern Europeans.	presence of association for rs2046748 (p = 2.5 × 10−3,OR = 1.82, 95%CI = 1.24–2.69)	sclerosis (PrMS)
rs6956703	0.223	7	154630460	intron	-	1000 Genomes	Association between DPP6 polymorphism and the risk of progressive multiple sclerosis in Northern and Southern Europeans.	This gene emerged as a candidate gene in a genome-wide association study (GWAS) performed in an Italian sample of PrMS and controls in which two SNPs located in the gene (rs6956703 and rs11767658) showed evidence of association (nominal p-value < 10−4).	progressive forms of multiple sclerosis (PrMS)

Supplementary Table 1 (Continued)

Functional regional SNPs (DSCAM)							
rs number	MAF in (CHB/HCB)	Chr	Location	Function	Function Prediction	Source	Reference
rs10706289	0.461	21	40011614	3‘UTR	-	1000 Genomes	No references
rs11390635	0.107	21	40012076 - 40012077	3‘UTR	-	1000 Genomes	No references
rs11451228	0.141	21	40012907 - 40012908	3‘UTR	-	1000 Genomes	No references
rs16999204	0.131	21	40044139	cds-synon	Splicing(ESE or ESS)	1000 Genomes	No references
rs2297263	0.087	21	40083927	cds-synon	Splicing(ESE or ESS)	1000 Genomes	No references
rs2297267	0.131	21	40187255	cds-synon	Splicing(ESE or ESS)	1000 Genomes	No references
rs2297270	0.238	21	40353703	missense	Splicing(ESE or ESS)、nsSNP	1000 Genomes	No references
rs2837371	0.33	21	40011216	3‘UTR	-	1000 Genomes	No references
rs2837372	0.442	21	40011541	3‘UTR	-	1000 Genomes	No references
rs2837373	0.053	21	40011912	3‘UTR	-	1000 Genomes	No references
rs34336407	0.131	21	40312163	cds-synon	-	1000 Genomes	No references
rs4818107	0.136	21	40011485	3‘UTR	-	1000 Genomes	No references
rs4818108	0.461	21	40011856	3‘UTR	-	1000 Genomes	No references
rs60564669	0.107	21	40012740	3‘UTR	-	1000 Genomes	No references
rs7275460	0.121	21	40075131	cds-synon	-	1000 Genomes	No references
rs73221332	0.053	21	40011913	3‘UTR	-	1000 Genomes	No references
rs73221333	0.383	21	40012010	3‘UTR	-	1000 Genomes	No references
rs9975082	0.461	21	40847002	5‘UTR	TFBS	1000 Genomes	No references

Continued									
Validated Hot SNPs (DSCAM)									
rs number	MAF in (CHB/HCB)	Chr	Location	Function	Function Prediction	Source	Reference	Comment	Disease
rs2222973		21	40461957	intron	-	1000 Genome	Analysis of Single Nucleotide Polymorphism in Adolescent Idiopathic Scoliosis in Korea: For Personalized Treatment.	closely associated with AIS severity	Adolescent Idiopathic Scoliosis

Supplementary Table 1 (Continued)

Functional regional SNPs (LSAMP)							
rs number	MAF in (CHB/HCB)	Chr	Location	Function	Function Prediction	Source	Reference
rs1046208	0.277	3	115809416	3‘UTR	-	1000 Genomes	No references
rs1062118	0.112	3	115803713	3‘UTR	-	1000 Genomes	No references
rs11718869	0.092	3	115809609	3‘UTR	-	1000 Genomes	No references
rs11719103	0.131	3	115804452	3‘UTR	-	1000 Genomes	No references
rs144940159	0.136	3	115808108	3‘UTR	-	1000 Genomes	No references
rs2289270	0.112	3	115810070	3‘UTR	miRNA(Sanger)	1000 Genomes	No references
rs2289271	0.359	3	115809913	3‘UTR	-	1000 Genomes	No association
rs28636502	0.058	3	115809664	3‘UTR	-	1000 Genomes	No references
rs2972475	0.408	3	115809555	3‘UTR	-	1000 Genomes	No references
rs3214679	0.364	3	115809962 - 115809963	3‘UTR	-	1000 Genomes	No references
rs60436476	0.131	3	115802527	3‘UTR	-	1000 Genomes	No references
rs73858074	0.058	3	115809657	3‘UTR	-	1000 Genomes	No references

rs938523	0.175	3	115803930	3‘UTR	-	1000 Genomes	No references
rs9876745	0.112	3	115810028	3‘UTR	-	1000 Genomes	No references

Continued

Validated Hot SNPs (LSAMP)

rs number	MAF in (CHB/HCB)	Chr	Location	Function	Function Prediction	Source	Reference	Comment	Disease
rs2918213	0.291	3	115833592	intron	-	1000 Genomes	Association of limbic system-associated membrane protein (LSAMP) to male completed suicide.	Chi square test revealed four allelic variants (rs2918215, rs2918213, rs9874470 and rs4821129) located in the intronic region of the gene to be associated with suicide.	suicide
rs2918215	0.214	3	115828629	intron	-	1000 Genomes	Association of limbic system-associated membrane protein (LSAMP) to male completed suicide.	Chi square test revealed four allelic variants (rs2918215, rs2918213, rs9874470 and rs4821129) located in the intronic region of the gene to be associated with	suicide

rs9874470	0.252	3	116246801	intron	-	1000 Genomes	Association of limbic system-associated membrane protein (LSAMP) to male completed suicide.	Chi square test revealed four allelic variants (rs2918215, rs2918213, rs9874470 and	suicide
								rs4821129) located in the intronic region of the gene to be associated with suicide.	

Supplementary Table 1 (Continued)

Functional regional SNPs (MX2)							
rs number	MAF in (CHB/HCB)	Chr	Location	Function	Function Prediction	Source	Reference
rs2301803	0.204	21	41399249	cds-synon	Splicing(ESE or ESS) 、Splicing(abolish domain)	1000 Genomes	No references
rs28425	0.461	21	41372958	5‘UTR	-	1000 Genomes	Evolutionary analysis identifies an MX2 haplotype associated with natural resistance to HIV-1 infection.
rs398206	0.184	21	41370109	5‘UTR	-	1000 Genomes	No references
rs398800	0.184	21	41370285	5‘UTR	-	1000 Genomes	No references

Continued									
Validated Hot SNPs (MX2)									
rs number	MAF in (CHB/HCB)	Chr	Location	Function	Function Prediction	Source	Reference	Comment	Disease
No	No	No	No	No	No	No	No	No	No

Supplementary Table 1 (Continued)

Functional regional SNPs (PRKG1)							
rs number	MAF in (CHB/HCB)	Chr	Location	Function	Function Prediction	Source	Reference
rs10128363	0.228	10	52184875	3‘UTR	-	1000 Genomes	No references
rs1045767	0.131	10	51695648	3‘UTR	miRNA(miRanda)	1000 Genomes	No references
rs13499	0.291	10	52297965	3‘UTR	-	1000 Genomes	No association
rs1881597	0.32	10	52294057	3‘UTR	miRNA(miRanda)	1000 Genomes	Interactions among genetic variants from contractile pathway of vascular smooth muscle cell in essential hypertension susceptibility of Chinese Han population.
rs1910548	0.393	10	52184900	3‘UTR	-	1000 Genomes	No references
rs35361017	0.383	10	50991328 - 50991329	5‘UTR	-	1000 Genomes	No references
rs5784918	0.306	10	52294153 - 52294154	3‘UTR	-	1000 Genomes	No references

Continued

Validated Hot SNPs (PRKG1)									
rs number	MAF in (CHB/HCB)	Chr	Location	Function	Function Prediction	Source	Reference	Comment	Disease
rs1881597	0.32	10	52294057	intron	-	1000 Genomes	Interactions among genetic variants from contractile pathway of vascular smooth muscle cell in essential hypertension susceptibility of Chinese Han population.		essential hypertensio n
rs1904694	0.393	10	51145734	intron	-	1000 Genomes	cGMP-Dependent Protein Kinase 1 Polymorphisms Underlie Renal Sodium Handling Impairment.	PRKG1 risk haplotype GAT (rs1904694,	Renal Sodium Handling

								rs7897633, rs7905063, respectively) associates with a rightward shift of the pressure–natriuresis curve (0.017±0.004 μEq/mm Hg per minute) compared with the ACC (0.0013±0.003 μEq/mm Hg per minute; P=0.001).	Impairment
rs7897633	0.476	10	51197961	intron	-	1000 Genomes	cGMP-Dependent Protein Kinase 1 Polymorphisms Underlie Renal Sodium Handling Impairment.	PRKG1 risk haplotype GAT (rs1904694, rs7897633, rs7905063, respectively) associates with a rightward shift of the pressure–natriuresis	Renal Sodium Handling Impairment

								curve (0.017±0.004 μEq/mm Hg per minute) compared with the ACC (0.0013±0.003 μEq/mm Hg per minute; P=0.001)	
rs7905063	0.476	10	51204830	intron	-	1000 Genomes	cGMP-Dependent Protein Kinase 1 Polymorphisms Underlie Renal Sodium Handling Impairment.	PRKG1 risk haplotype GAT (rs1904694, rs7897633, rs7905063, respectively) associates with a rightward shift of the pressure–natriuresis curve (0.017±0.004 μEq/mm Hg per minute) compared with the ACC (0.0013±0.003 μEq/mm Hg per minute; P=0.001).	Renal Sodium Handling Impairment

Supplementary Table 1 (Continued)

Functional regional SNPs (PTPRT)						
rs number	MAF in (CHB/HCB)	Location	Function	Function Prediction	Source	Reference
rs1065155	0.194	42077716	3‘UTR	miRNA(miRanda)	1000 Genomes	No association
rs1883374	0.165	42098028	3‘UTR	-	1000 Genomes	No references
rs1884039	0.194	42077597	3‘UTR	miRNA(miRanda)	1000 Genomes	No references
rs1884040	0.194	42077854	3‘UTR	miRNA(miRanda)	1000 Genomes	No references
rs2016647	0.053	42085839	cds-synon	Splicing(ESE or ESS)	1000 Genomes	No references
rs2144011	0.364	42074487	3‘UTR	miRNA(miRanda)	1000 Genomes	No references
rs2425516	0.374	42677960	cds-synon	-	1000 Genomes	No references
rs2664587	0.369	42074172	3‘UTR	miRNA(miRanda)	1000 Genomes	No references
rs2867655	0.15	43189649	missense	Splicing(ESE or ESS)、nsSNP	1000 Genomes	No association
rs3787281	0.272	42075482	3‘UTR	-	1000 Genomes	No references
rs3838030	0.165	42072975	3‘UTR	-	1000 Genomes	No references
rs45474194	0.058	42077233	3‘UTR	miRNA(miRanda)	1000 Genomes	No references
rs55683344	0.058	42079703	3‘UTR	miRNA(miRanda)	1000 Genomes	No references
rs6065432	0.058	42079594	3‘UTR	miRNA(miRanda)	1000 Genomes	No references
rs6102662	0.058	42077204	3‘UTR	miRNA(miRanda)	1000 Genomes	No references
rs6102663	0.471	42079069	3‘UTR	miRNA(miRanda)	1000 Genomes	No references
rs6130027	0.359	42078591	3‘UTR	miRNA(miRanda)	1000 Genomes	No references
rs67506870	0.058	42080132	3‘UTR	-	1000 Genomes	No references
rs72626509	0.214	42077531	3‘UTR	-	1000 Genomes	No references
rs7263976	0.354	42079297	3‘UTR	miRNA(miRanda)	1000 Genomes	No references
rs73269502	0.17	42076612	3‘UTR	-	1000 Genomes	No references

rs73271408	0.175	42079224	3‘UTR	-	1000 Genomes	No references
rs877431	0.058	42077655	3‘UTR	miRNA(miRanda)	1000 Genomes	No references

Continued

Validated Hot SNPs (PTPRT)							
rs number	MAF in (CHB/HCB)	Function	Function Prediction	Source	Reference	Comment	Disease
rs11086843	0.354	intron	-	1000 Genomes	Genome-wide association study of rheumatoid arthritis in the Spanish population: KLF12 as a risk locus for rheumatoid arthritis susceptibility.	rs6030267 (P = 4.08 × 10−5) and rs11086843 (P = 2.07 × 10−6)	rheumatoid arthritis
rs2866943	0.058	intron	-	1000 Genomes	The Functional Variant in the 3'UTR of PTPRT with the Risk of Esophageal Squamous Cell Carcinoma in a Chinese Population.		
rs3746539	0.466	3‘UTR	miRNA(miRanda)	1000 Genomes	Identification of group of hypertension-susceptibility genes		
rs6030267	0.044	intron	-	1000 Genomes	Genome-wide association study of rheumatoid arthritis in the Spanish population: KLF12 as a risk locus for rheumatoid arthritis susceptibility.	rs6030267 (P = 4.08 × 10−5) and rs11086843 (P = 2.07 × 10−6)	rheumatoid arthritis

Supplementary Table 1 (Continued)

Functional regional SNPs (ROBO2)						
rs number	MAF in (CHB/HCB)	Location	Function	Function Prediction	Source	Reference
rs3923745	0.335	77040244	5‘UTR	TFBS	1000 Genomes	No references
rs3923744	0.228	77040548	5‘UTR	TFBS	1000 Genomes	No references
rs11127602	0.18	77647169	3‘UTR	miRNA(miRanda)	1000 Genomes	No references
rs1031377	0.481	77647271	3‘UTR	miRNA(miRanda)	1000 Genomes	No association
rs1163748	0.296	77648792	3‘UTR	-	1000 Genomes	No references

rs1163750	0.476	77649291	3'UTR	-	1000 Genomes	No references

Continued							
Validated Hot SNPs (ROBO2)							
rs number	MAF in (CHB/HCB)	Function	Function Prediction	Source	Reference	Comment	Disease
No	No	No	No	No	No	No	No

Supplementary Table 1 (Continued)

Functional regional SNPs (STK32B)						
rs number	MAF in (CHB/HCB)	Location	Function	Function Prediction	Source	Reference
rs2369706	0.354	5437963	5'UTR	-	1000 Genomes	No references
rs2369707	0.354	5438101	5'UTR	-	1000 Genomes	No references
rs2369708	0.354	5438102	5'UTR	-	1000 Genomes	No references
rs3733180	0.413	5448397	3'UTR	-	1000 Genomes	No references
rs3733182	0.442	5446702	missense	Splicing(ESE or ESS)、nsSNP	1000 Genomes	No references
rs3774835	0.34	5438079	5'UTR	-	1000 Genomes	No references
rs3774836	0.34	5436601	5'UTR	-	Hapmap	No references
rs3774837	0.34	5436555	5'UTR	-	Hapmap	No references
rs3774838	0.34	5436517	5'UTR	-	Hapmap	No references
rs3774839	0.34	5436458	5'UTR	-	Hapmap	No references
rs3774840	0.34	5436402	5'UTR	-	Hapmap	No references
rs4689235	0.35	5437588	5'UTR	-	Hapmap	No references
rs4689236	0.29	5437697	5'UTR	-	Hapmap	No references
rs4689237	0.37	5437903	5'UTR	-	Hapmap	No references
Continued						

Validated Hot SNPs (STK32B)							
rs number	MAF in (CHB/HCB)	Function	Function Prediction	Source	Reference	Comment	Disease
No	No	No	No	No	No	No	No

Supplementary Table 1 (Continued)

Functional regional SNPs (TMEM132D)						
rs number	MAF in (CHB/HCB)	Location	Function	Function Prediction	Source	Reference
rs10773594	0.44	129075012	cds-synon	Splicing(ESE or ESS)	Hapmap	No references
rs2292723	0.21	129071915	3'UTR	miRNA(miRanda)	Hapmap	No references
rs492759	0.208	129072340	3'UTR	miRNA(miRanda)	1000 Genomes	No references
rs60962336	0.183	129081795	cds-synon	Splicing(ESE or ESS)	1000 Genomes	No association
rs61944776	0.258	129073202	3'UTR	miRNA(miRanda)	1000 Genomes	No references
rs73159540	0.121	129074876	missense	-	1000 Genomes	No association
rs77363876	0.214	129081924	cds-synon	-	1000 Genomes	No association
rs79031518	0.131	129081861	cds-synon	-	1000 Genomes	No association

Continued							
Validated Hot SNPs (TMEM132D)							
rs number	MAF in (CHB/HCB)	Function	Function Prediction	Source	Reference	Comment	Disease
rs11060369	0.418	intron	-	1000 Genomes	1. Replication and meta-analysis of TMEM132D gene variants in panic disorder.	(rs7309727 and rs11060369) located in intron 3 of TMEM132D to be associated with PD	panic disorder (PD)

					2. Polymorphisms in the TMEM132D region are associated with panic disorder in HLA-DRB1*13:02-negative individuals of a Japanese population.		
					3. TMEM132D, a new candidate for anxiety phenotypes: evidence from human and mouse studies.	stage 1: allelic model P=0.0001, Armitage test P=0.0001	
rs4759997	0.2	intron	-	1000 Genomes	Polymorphisms in the TMEM132D region are associated with panic disorder in HLA-DRB1*13:02-negative individuals of a Japanese population.	P=5.02×10−6, odds ratio=1.50	panic disorder (PD)
rs7309727	0.34	intron	-	1000 Genomes	1. Replication and meta-analysis of TMEM132D gene variants in panic disorder.	rs7309727 and rs11060369) located in intron 3 of TMEM132D to be associated with PD.	panic disorder (PD)
					2. Polymorphisms in the TMEM132D region are associated with panic disorder in HLA-DRB1*13:02-negative individuals of a Japanese population.	rs7309727 and rs11060369 as susceptibility variants for PD	panic disorder (PD)
					3. TMEM132D, a new candidate for anxiety phenotypes: evidence from human and mouse studies.	allelic test: P=5.1e−7, Armitage test of trend: P=7.726e−7	

Supplementary Table 1 (Continued)

Functional regional SNPs (TMPRSS3)						
rs number	MAF in (CHB/HCB)	Location	Function	Function Prediction	Source	Reference
rs13047838	0.5	42372760	3‘UTR	Splicing(ESE or ESS)、Splicing(abolish domain)、miRNA(miRanda)	1000 Genomes	No association

rs2839500	0.388	42383058	missense	Splicing(ESE or ESS)、nsSNP	1000 Genomes	No association
rs2839501	0.233	42385528	cds-synon	Splicing(ESE or ESS)	1000 Genomes	No association
rs928302	0.175	42389975	missense	Splicing(ESE or ESS)、nsSNP	1000 Genomes	No association

Continued

Validated Hot SNPs (TMPRSS3)

rs number	MAF in (CHB/HCB)	Function	Function Prediction	Source	Reference	Comment	Disease
rs1078272	0.44	intron	-	Hapmap	Functional variants at the 21q22.3 locus involved in breast cancer progression identified by screening of genome-wide estrogen response elements.	Three single-nucleotide polymorphisms (SNPs) were found to be significantly associated with breast cancer progression, of which two (rs2839494 and rs1078272) were associated with estrogen response elements (EREs).	breast cancer progression
rs2251362	0.34	intron	-	Hapmap	Functional variants at the 21q22.3 locus involved in breast cancer progression identified by screening of genome-wide estrogen response elements.	be significantly associated with breast cancer progression.	breast cancer progression
rs2839494	0.44	intron	-	Hapmap	Functional variants at the 21q22.3 locus involved in breast cancer progression identified by screening of genome-wide estrogen response elements.	Three single-nucleotide polymorphisms (SNPs) were found to be significantly associated with breast cancer progression, of which two (rs2839494 and rs1078272) were associated with estrogen response elements (EREs).	breast cancer progression

Supplementary Table 1 (Continued)

Functional regional SNPs (ZMAT4)						
rs number	MAF in (CHB/HCB)	Location	Function	Function Prediction	Source	Reference
rs72632903	0.068	40884538	missense	-	1000 Genomes	No reference

Continued							
Validated Hot SNPs (ZMAT4)							
rs number	MAF in (CHB/HCB)	Function	Function Prediction	Source	Reference	Comment	Disease
No	No	No	No	No	No	No	No
