# Supplementary material for: Sporadic Parkinson’s Disease Potential Risk Loci Identified in Han Ancestry of Chinese Mainland
Source: Front Aging Neurosci. 2021 Jan 12;12:603793. doi: 10.3389/fnagi.2020.603793 (PMC7835639; doi:10.3389/fnagi.2020.603793)
Supplement: Supplementary file 2 [file Data_Sheet_2.PDF]

Supplementary Table 2 Primers used in 384-wells PCR reactions

Extension products	SNP_ID	2nd-PCR Primer	1st-PCR Primer	AMP_LEN	UP_CONF	MP_CONF	Tm(NN)
COL5A2	rs10197596	ACGTTGGATGATAGTGCCCCATGATATCCC	ACGTTGGATGTAATTAGGGCCCTCCTGGTG	114	97.8	71.6	48.4
DMRT2	rs12002058	ACGTTGGATGTCGGCTTTTCGGTAGCTCTG	ACGTTGGATGGGAGAGGAAGTAAAAGTCTG	119	95.7	71.6	48.1
COL5 A2	rs11186	ACGTTGGATGAACATGTAGGTCCCCTTGTG	ACGTTGGATGGGTCCCAGCTGTTTATTTTA	118	90.8	71.6	47.1
FGF10/MRPS30	rs13153459	ACGTTGGATGAAGAGCCTACTGTATGACTG	ACGTTGGATGATTGAAAGATCCCCATGGGC	107	98.4	71.6	47.5
CYP1B1/C2orf58	rs163090	ACGTTGGATGATCAGTTAAGGCAGGAACCG	ACGTTGGATGTGTATTGTTAGGCCCCTGAG	113	99.3	71.6	45.2
PLEKHN1	rs28499371	ACGTTGGATGGTAGTGGAAGAGCTTGTTGG	ACGTTGGATGTTGTCCCCAGAGAGGACAG	118	94.7	71.6	54.9
PLEKHN1	rs3829738	ACGTTGGATGTGTGTCTGTGCCTGCCTCTG	ACGTTGGATGCTTCTTGGAGAGCAAGTAGG	93	94.2	71.6	56.8
C3orf67/LOC339902	rs6783485	ACGTTGGATGAAGAATTGCAGCCTCTTGCC	ACGTTGGATGCCACTCCAAGCATATGTCAG	104	99.9	71.6	45.5
FLJ35379/LOC100132423	rs61959631	ACGTTGGATGCTGACTCTCTGATGACAAGC	ACGTTGGATGCTGCTGCTTTTTGGTGACTG	93	99.8	71.6	52
DMRT2	rs2279984	ACGTTGGATGGTAGTTTGCATACACGTCGC	ACGTTGGATGGTTTTTTCAGCGTCTTCCCC	101	99.8	71.6	45.5
COL5A2	rs6434312	ACGTTGGATGGGTGACAAAGGTGATCATGG	ACGTTGGATGGACCCTGAAGACCAGTAAAG	96	99.9	71.6	46.7
ANXA1/LOC100130911	rs10746953	ACGTTGGATGGGGCAAGCAGAATCTGAAAG	ACGTTGGATGGAAAAGGGAGGTGAAACGAG	98	100	71.6	48.8
FNDC3B	rs2270568	ACGTTGGATGAAAGCTTTGTCCGGCAATGG	ACGTTGGATGGGCAAGAGGACAAGGAAACG	109	97.9	71.6	48.7
BARX1/PTPDC1	rs10993010	ACGTTGGATGCCTTTTGATCTCTCTTCGTG	ACGTTGGATGCGAGTGCTAAGTCTTTGCAG	88	98	71.6	47.3
PDE10A	rs880121	ACGTTGGATGTGGAACTCACCTGCTCTCTG	ACGTTGGATGATCCTGAGATGGAAGGTAGC	111	98.1	71.6	49.4
BARX1	rs11793856	ACGTTGGATGAGCAGCTTACTGAGCAGGAG	ACGTTGGATGTATACCGCCCTCAGTCCTC	119	93.2	71.6	46.7
FNDC3B	rs7652177	ACGTTGGATGGGGTTTATCATAATCATCC	ACGTTGGATGTTGCTGTACTGGTCTTCTCG	104	87.9	71.6	46.2
DMRT2/SMARCA2	rs80315856	ACGTTGGATGGGACTCAGAGATGAGTTTGG	ACGTTGGATGTATCTGGCCCCAAGTTTCTG	97	100	71.6	52
ZNF396/INO80C	rs1362858	ACGTTGGATGTTAGGTTACGATAGGTTAC	ACGTTGGATGGAAGACCTAAATAACTGGAG	95	82.9	71.6	46.9
BARX1	rs191789925	ACGTTGGATGTGATTGAGGAGATCCTCACG	ACGTTGGATGACGCCGAACTTCAGCAGCTC	114	93.6	71.6	69.3
TSG1/MANEA	rs9445283	ACGTTGGATGAGGACATGTAGCTCTGTGAC	ACGTTGGATGCCTTTGCAGATACTATATGGC	111	94.1	71.6	45.5
PLEKHN1	rs3829740	ACGTTGGATGAGAGGCAGGCACAGACACAG	ACGTTGGATGAGTTCCTCAGTGCCATGCAG	103	93	71.6	62.3
FLJ23172/FNDC3B	rs73180248	ACGTTGGATGCAGCAGCATGTAAGATGAAG	ACGTTGGATGTGCATAGCTACCTAGAGGAG	116	97.6	71.6	46.5

Continued							
LOC285194/IGSF11	rs1879553	ACGTTGGATGCCCTCAGTTTCTCATTATCC	ACGTTGGATGTCAAGATGGAATCCTACAAG	99	93	71.6	48.2
COL5A2	rs11691604	ACGTTGGATGTTAAAGGGTGTGTGTCTGGG	ACGTTGGATGTAAGAAAGGCTGTTAGAAG	94	82.1	71.6	51.5
PAQR3/ARD1B	rs201453169	ACGTTGGATGGAAAGGTTATGCGAGGTAAG	ACGTTGGATGAGGGCATATTTACCAGCTCC	106	98.4	71.6	47.6
ATPBD4/LOC100288892	rs17534343	ACGTTGGATGTAGAAGTGACTTTCCTGCTG	ACGTTGGATGACTTTGAGCCAGATCCTGCC	100	97.1	71.6	47.8
MDGA2	rs12590500	ACGTTGGATGACAAGAAAGCTGCATCTCCC	ACGTTGGATGACGCTGTGAAGAGTCTTTCC	111	99.5	71.6	48.2
MDGA2	rs9323124	ACGTTGGATGCACCTAGTTTTTGGCCACTG	ACGTTGGATGGAATGGGTGAAGTAGGGAAC	97	100	71.6	54.1
ZFP64/TSHZ2	rs863108	ACGTTGGATGTCACTGCTGTGCTAAAATGG	ACGTTGGATGCTCTTCCTCCTTTATGTTCC	103	97.1	71.6	46

Supplementary Table 2 *(Continued)*

PcGC	PWARN	UEP_DIR	UEP_MASS	UEP_SEQ	EXT1_CALL (SNP genotype)	EXT1_MASS	EXT1_SEQ (Extension primers)	EXT2_CALL (SNP genotype)
60	Dh	F	4531	ATCCCCAAGAGCAGC	G	4818.2	ATCCCCAAGAGCAGCG	T
60	D	R	4660.1	GACAAGCGACAGGGA	T	4931.3	GACAAGCGACAGGGAA	C
50	d	R	4783.1	TCCCCTTGTGTCTCAA	C	5070.3	TCCCCTTGTGTCTCAAG	A
60	D	R	4897.2	tTGACTGATCCAGGGC	C	5184.4	tTGACTGATCCAGGGCG	A
50	Dg	F	4992.3	GGGGATATGATGGCTT	A	5263.5	GGGGATATGATGGCTTA	T
68.8	D	R	5147.3	gGTCCCCGCTTCGAGCA	T	5418.6	gGTCCCCGCTTCGAGCAA	C
66.7	Ds	F	5362.5	TCTGACCCTCGCTCCTGC	C	5609.7	TCTGACCCTCGCTCCTGCC	T
50	D	R	5523.6	CCCGTAGTCTTAGAGAAG	G	5770.8	CCCGTAGTCTTAGAGAAGC	A
68.8	D	R	5565.6	ccGAGTGTGCCCAGGGAG	G	5812.8	ccGAGTGTGCCCAGGGAGC	A
53.3	d	F	5739.7	ccctCCTGTTTGCAAAGGC	A	6010.9	ccctCCTGTTTGCAAAGGCA	T
60	D	R	5933.9	gtggTCATGGAGACCGAGG	G	6181	gtggTCATGGAGACCGAGGC	A
42.1	D	R	5940.9	AGAATCTGAAAGTGGTGGA	T	6212.1	AGAATCTGAAAGTGGTGGAA	C
52.9	D	F	6111	ccaGCTCGAAACGACATTGG	C	6358.2	ccaGCTCGAAACGACATTGGC	T

Continued								
35	d	R	6149	TCGTGTTAGAAACAAAATGC	G	6396.2	TCGTGTTAGAAACAAAATGCC	A
52.9	ds	F	6324.1	ccgtAGCATCTGGCTTCTTCC	C	6571.3	ccgtAGCATCTGGCTTCTTCCC	G
47.1	d	R	6522.3	ggcaAAGGATGCAGAGAAACC	G	6769.5	ggcaAAGGATGCAGAGAAACCC	C
27.3	D	F	6732.4	ATACCATTTTATGGAATGTCAA	C	6979.6	ATACCATTTTATGGAATGTCAAC	G
47.4	d	R	6917.5	gggTGAGTTTGGTTGGAGCTGA	G	7164.7	gggTGAGTTTGGTTGGAGCTGAC	T
34.8		R	7126.7	GGTTACGATAGGTTACAATTAAG	C	7413.9	GGTTACGATAGGTTACAATTAAGG	A
84.2	d	R	7284.7	cttcaCAAGGGCGCCGCGCCCGCA	T	7555.9	cttcaCAAGGGCGCCGCGCCCGCAA	C
29.2		F	7334.8	CTATCTATCTCAGTTAGGTAAAAA	A	7606	CTATCTATCTCAGTTAGGTAAAAAA	T
73.7	DH	R	7490.8	ggtgaGCTCGAGGGCGTGGGTCCA	G	7738	ggtgaGCTCGAGGGCGTGGGTCCAC	C
30.4	d	F	7695	tcAGATGAAGTATATGTAGTTCTCA	A	7966.2	tcAGATGAAGTATATGTAGTTCTCAA	G
30.4	D	R	7819.1	tccGTTTCTCATTATCCATTTTGCTT	G	8066.3	tccGTTTCTCATTATCCATTTTGCTTC	A
50	g	R	7925.2	gggacGGGTTAGGGAGGGGAAAATA	T	8196.4	gggacGGGTTAGGGAGGGGAAAATAA	C
23.1	d	F	8025.3	ATAACTTTAAGCAGATTTAAAGGTAA	A	8296.5	ATAACTTTAAGCAGATTTAAAGGTAAA	T
28		F	8115.3	gGAAAGAGAATATGGCAAGTTTAATA	A	8386.5	gGAAAGAGAATATGGCAAGTTTAATAA	T
25.9		F	8247.4	CTTCATTTATGATGCTACAGTTATAAA	C	8494.6	CTTCATTTATGATGCTACAGTTATAAAC	A
47.8	d	R	8554.6	gttgaCCACTGTAAAGCTATTTCCTGGC	T	8825.8	gttgaCCACTGTAAAGCTATTTCCTGGCA	A
21.7	ds	F	8670.7	cttcgGTGCTAAAATGGAAAAAAAAAAA	A	8941.9	cttcgGTGCTAAAATGGAAAAAAAAAAAA	T

Supplementary Table 2 *(Continued)*

EXT2_CALL (SNP genotype)	EXT2_MASS	EXT2_SEQ (Extension primers)
T	4858.1	ATCCCCAAGAGCAGCT
C	4947.3	GACAAGCGACAGGGAG
A	5110.2	TCCCCTTGTGTCTCAAT

Continued		
A	5224.3	tTGACTGATCCAGGGCT
T	5319.4	GGGGATATGATGGCTTT
C	5434.6	gGTCCCCGCTTCGAGCAG
T	5689.6	TCTGACCCTCGCTCCTGCT
A	5850.7	CCCGTAGTCTTAGAGAAGT
A	5892.7	ccGAGTGTGCCCAGGGAGT
T	6066.8	ccctCCTGTTTGCAAAGGCT
A	6261	gtggTCATGGAGACCGAGGT
C	6228.1	AGAATCTGAAAGTGGTGGAG
T	6438.1	ccaGCTCGAAACGACATTGGT
A	6476.1	TCGTGTTAGAAACAAAATGCT
G	6611.3	ccgtAGCATCTGGCTTCTTCCG
C	6809.5	ggcaAAGGATGCAGAGAAACCG
G	7019.6	ATACCATTTTATGGAATGTCAAG
T	7188.7	gggTGAGTTTGGTTGGAGCTGAA
A	7453.8	GGTTACGATAGGTTACAATTAAGT
C	7571.9	cttcaCAAGGGCGCCGCGCCCGCAG
T	7661.9	CTATCTATCTCAGTTAGGTAAAAAT
C	7778.1	ggtgaGCTCGAGGGCGTGGGTCCAG
G	7982.2	tcAGATGAAGTATATGTAGTTCTCAG
A	8146.2	tccGTTTCTCATTATCCATTTTGCTTT
C	8212.4	gggacGGGTTAGGGAGGGGAAAATAG
T	8352.4	ATAACTTTAAGCAGATTTAAAGGTAAT

Continued		
T	8442.4	gGAAAGAGAATATGGCAAGTTTAATAT
A	8518.6	CTTCATTTATGATGCTACAGTTATAAAA
A	8881.7	gttgaCCACTGTAAAGCTATTTCCTGGCT
T	8997.8	TCTGACCCTCGCTCCTGCT
