# Supplementary material for: Sporadic Parkinson’s Disease Potential Risk Loci Identified in Han Ancestry of Chinese Mainland
Source: Front Aging Neurosci. 2021 Jan 12;12:603793. doi: 10.3389/fnagi.2020.603793 (PMC7835639; doi:10.3389/fnagi.2020.603793)
Supplement: Supplementary file 3 [file Data_Sheet_3.PDF]

系 统 生 物 学 技 术 服 务

Your Own Laboratory-----------您的专属实验室

艾康健生物

SNP 分型检测报告

武汉艾康健生物科技有限公司

Wuhan Icongene Biological Technology Co.,Ltd.

目录

1　项目信息 3

1.1　合同编号：BMSW20170807LHY0063 3

1.2　项目委托方：南昌大学附属第一医院 3

1.3　项目承接方：武汉艾康健生物科技有限公司 3

1.4　项目内容：SNP 分型检测 3

2　实验结果及数据 4

2.1　Massarray 实验数据分析报告 4

2.2　实验结论 4

3　实验流程与方法 5

3.1　Massarray SNP 分型实验流程说明 5

3.2　Massarray SNP 分型实验操作步骤 5

3.2.1　引物设计及合成、稀释 5

3.2.2　DNA 提取 8

3.2.3　Agena MassArray 系统基因分型步骤 9

3.3　数据分析流程说明 13

3.4　SNP genotype 分析方法介绍 13

4 附录 实验仪器和设备、试剂或软件 14

1.　主要实验仪器和设备 14

2.　主要试剂或软件 15

1 项目信息

1.1 合同编号：BMSW20170807LHY0063

1.2 项目委托方：南昌大学附属第一医院

项目联系人：王博　　　　电话：

所属部门：　　　　　　　电子信箱：

1.3 项目承接方：武汉艾康健生物科技有限公司

项目负责人：张伟伟　　　电话：15171429779

所属部门：总部　　　　　电子信箱：272851970@qq.com

1.4 项目内容：SNP 分型检测

实验平台：Agena 平台　　　样本类型：

样本物种信息：　　　　　　样本数量：1016

2 实验结果及数据

2.1 Massarray 实验数据分析报告

- 实验数据：

原始结果，请查阅本报告附件： 原始结果.rar，

提示：若本地电脑无法正常打开散点图，可采用 PictureManager2003 视图软件查看，该款软件下载地址: http://pan.baidu.com/s/1o8c2b0I 密码: yr75，按照提示进行安装即可。

SNP 分型数据结果，请查阅本报告附件：LHY0063_SNP 检测结果.rar

- 实验结果：对于 30 个待检的 SNP 位点，数据结果如下列表：

SNP 位点	检出率	备注	SNP 位点	检出率	备注
rs201453169	99%		rs9323124	99%	
rs6783485	99%		rs10197596	99%	
rs10746953	99%		rs10993010	99%	
rs3829738	99%		rs163090	99%	
rs6434312	99%		rs12590500	99%	
rs880121	99%		rs2279984	99%	
rs11793856	99%		rs2270568	98%	
rs12002058	99%		rs61959631	98%	
rs13153459	99%		rs1362858	98%	
rs80315856	99%		rs28499371	98%	
rs17534343	99%		rs7652177	98%	
rs9445283	99%		rs863108	98%	
rs73180248	99%		rs3829740	98%	
rs11186	99%		rs11691604	97%	
rs1879553	99%		rs191789925	51%	

2.2 实验结论

实验已经按照计划完成，所有实验数据均为真实、可靠数据，并且在附属文档中有所有的原始数据结果，整体实验过程中并没有因为实验操作失误导致的数据结果不准确，符合总结报告的要求。

3 实验流程与方法

3.1 Massarray SNP 分型实验流程说明

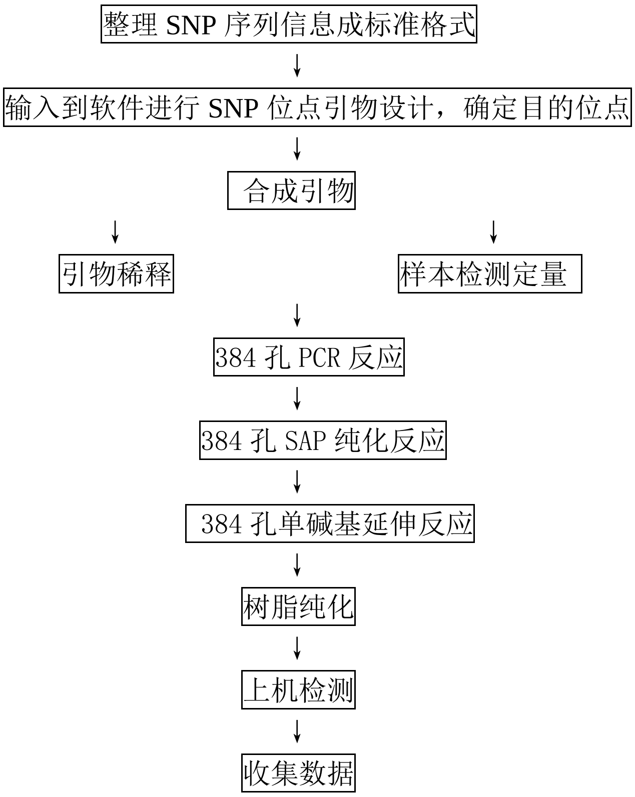

3.2 Massarray SNP 分型实验操作步骤

3.2.1 引物设计及合成、稀释

1. 基因序列的获取

1) 在 My agena 网站中注册成用户。

2) 在 NCBI 网页（http://www.ncbi.nlm.nih.gov/projects/SNP/）中输入该 SNP 位点的名称,按照 dbSNP batch reportor 格式显示（图 1）

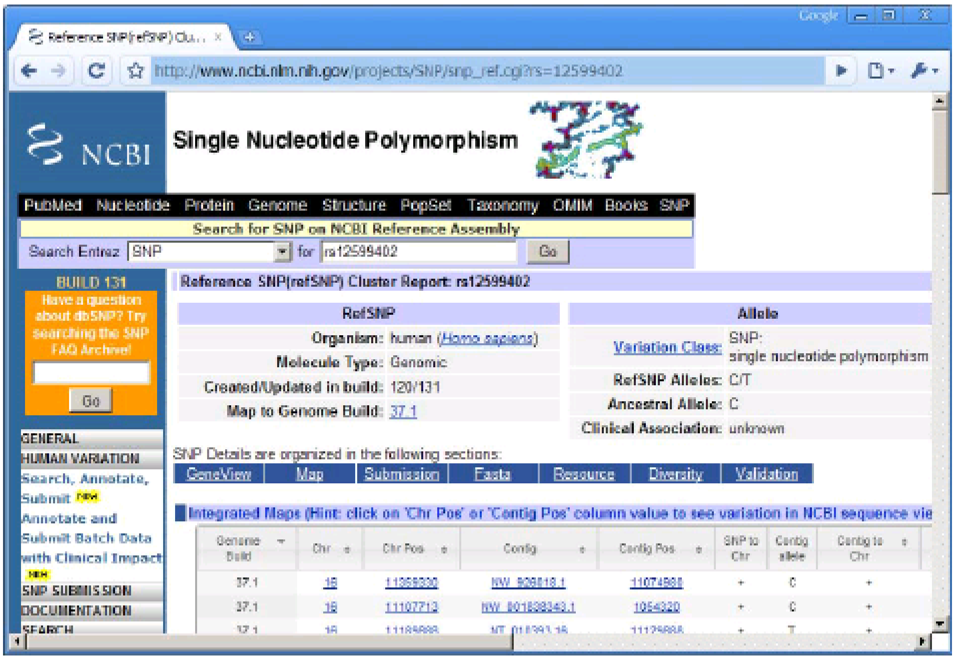

图 1 NCBI 上的 SNP 界面

3) 将 SNP 位点所在序列发送到 My agena 网站注册的邮箱中（图 2）

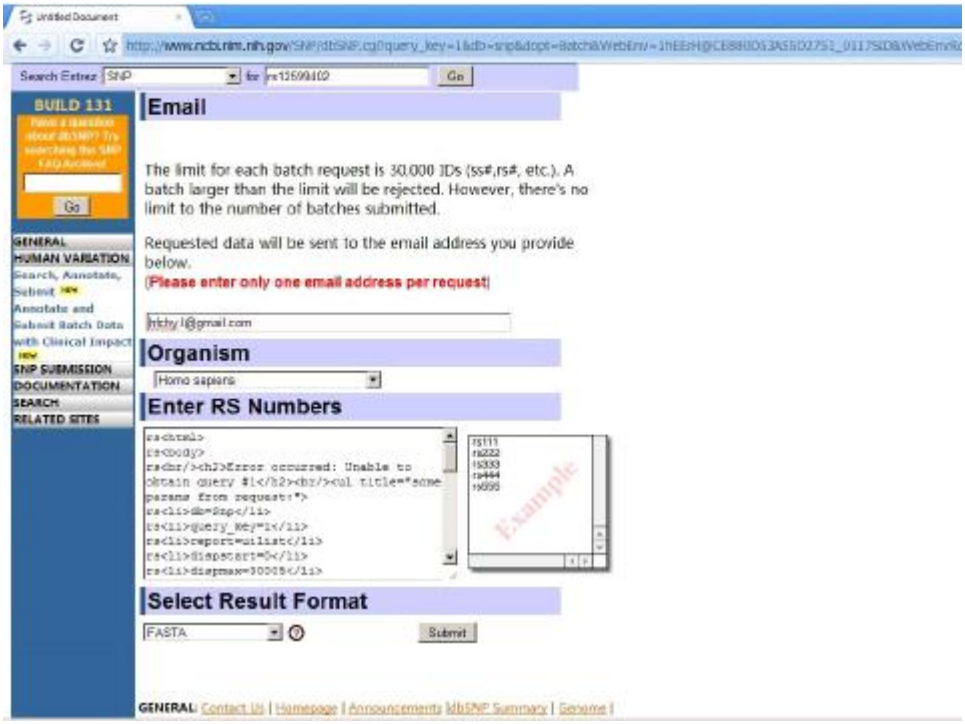

图 2 邮箱界面中的网址

4) 在 My agena 网站的 TOOLS 工具栏中选择 Genotyping。

5) 点击 RS format，在 Browse 按钮中选择 NCBI 网站发送到邮箱的文件。

6) 网站对序列的格式化完成后在 Sent to 栏中，选择 ProxSNP。

7) 开始 ProxSNP，点击 Begin Start。

8) 上述步骤完成后，在 Sent to 栏中选择 PreXTEND。

9) 开始 ProxSNP，点击 Begin Start。

10) 在产生的结果中，选择 OUTPUT，并把文件内容复制在新的文本文件.txt 中（图 3）

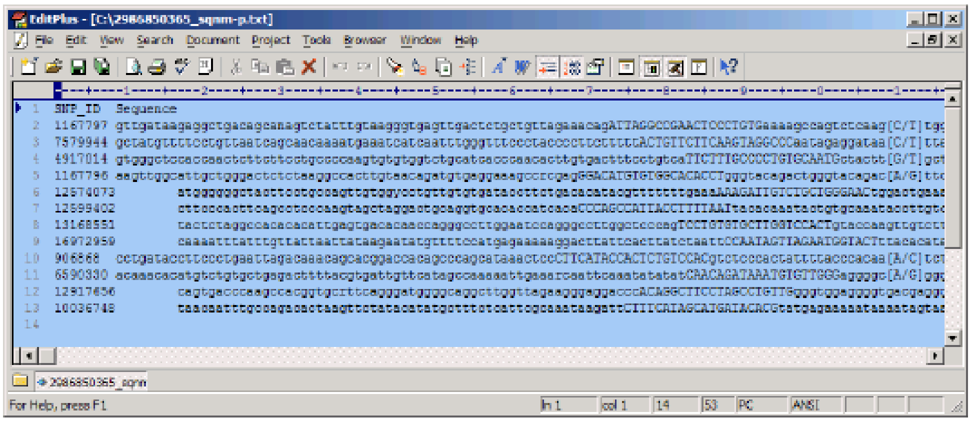

图 3 txt 文件内容

2. 结合文献并采用 AssayDesigner3.1 软件进行引物设计 PCR 反应和单碱基扩展引物，并交由生物公司合成。

1) 在软件 SNP Group 栏中选择 Browse 按钮，找到上述产生的 txt 文件。

2) 在 Aassy Design 栏中选择 SBE Mass Extend ,并在 SBE stop mix 栏中选择 iPlex，在 Mμltiplex Level 中按照实际情况选择不同的反应重数（图 4）。

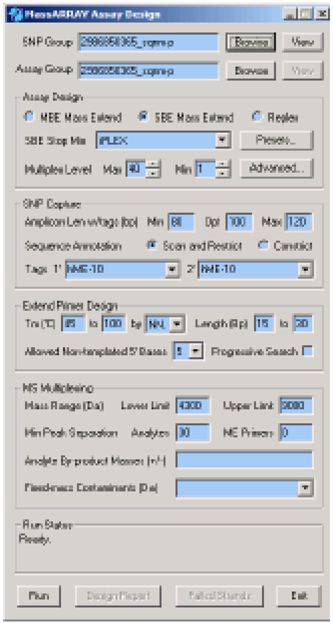

图 4　引物设计过程

3) SNP capture，Extend primer design，MASS Mμltiplexing 均选择默认参数。

4) 参数设定后，点击 Run。

5) 在 txt 文件目录相应位置找到产生的引物序列文件，设计的引物序列见 Primer 表格。

3. 引物稀释

1) PCR master mix 引物配置： 稀释单管 PCR master 至浓度 100 μM，加入去离子水混合所有单管 PCR master 使最终反应 PCR master mix 浓度为 0.5 μM。

2) EXTEND Mix 引物配置：稀释单管延伸引物至终浓度 500 μM，加入引物混合后使得各引物浓度为 8 μM、10 μM、15 μM。按照 DNA 合成产品使用说明计算该条引物分子量、质量数和摩尔数， 进而根据所需的浓度计算需加入去离子水的量。将混合好的单管延伸引物根据分子量大小，分别取（小于 6300Da）1 倍，（6300 Da 至 7200 Da）1.2 倍，（大于 7200 Da）1.5 倍体积量进行混合待用。

3.2.2 DNA 提取

使用成品化试剂盒，提取血样、组织、细胞、唾液中的 DNA。使用 NanoDrop2000 仪器进行 OD 值检测，1.25%琼脂糖凝胶电泳检测，DNA 质检合格，转移至 96 孔板，-20℃储存

备用。

3.2.3 Agena MassArray 系统基因分型步骤

分型原理：

通过 PCR 反应扩增出含有待检 SNP 位点的目的片段，然后用 SAP 酶去除 PCR 体系中剩余的脱氧核糖核苷三磷酸(dNTP)和引物,再加入单碱基延伸引物，其 3’末端碱基紧挨 SNP 位点，且与目的片段上的碱基完全互补，采用四种 ddNTP 替代 dNTP，这样，探针在 SNP 位点处仅延伸一个碱基，连接上的 ddNTP 与 SNP 位点的等位基因对应。用基质辅助激光解吸电离飞行时间质谱(MALDI-TOF MS) 检测延伸产物与未延伸引物间的分子量差异, 确定该点处碱基。

1. PCR 扩增反应

1) 取 1.5mlEP 管中配置 PCR master mix ，并振荡低速离心。反应组分见(表 1)：

表 1. PCR master mix 反应相关试剂配制组分

PCR master mix of Reagent	Conc.in 5μl	Volume (1rxn)
Water, HPLC grade	NA	1.850μl
PCR Buffer with 15mM $MgCl_2$	1.25x	0.625μl
$MgCl_2$ (25mM)	1.625mM	0.325μl
dNTP Mix(25 mM each)	500 μM	0.100μl
Primer Mix (500 nM each)	100uM	1.000μl
HotStar Taq(5U/μl)	0.5 U/rxn	0.100μl
Total	-	4.000μl
The final $MgCl_2$ concentration is 3.5 mM, 1.875 mM from the PCR buffer and 1.625 mM from the $MgCl_2$		

2) 选用 8 道或 12 道移液器，在 384 孔板的每个加样孔中加入 4μl PCR master mix，最后加入 1μl 模板 DNA（20ng/μl）混匀，小心盖上 384 孔封板膜，并压牢每个孔，防止 PCR 程序时出现蒸发等现象。1000 rpm 离心 1 minute。加样方式请参阅下图：

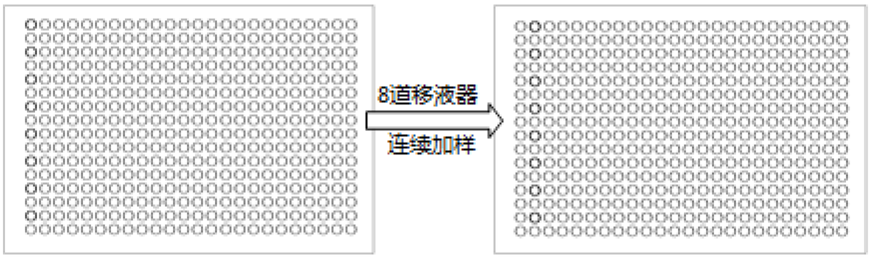

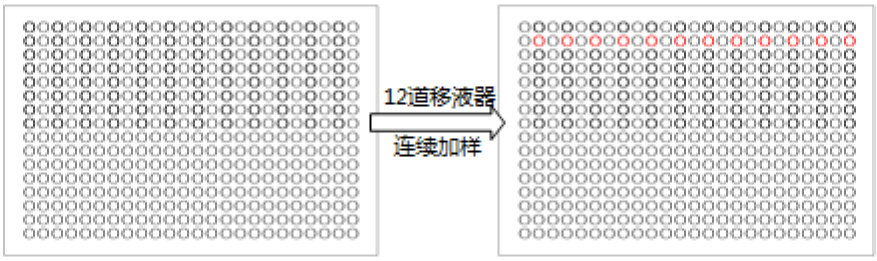

3) 设置 如下 PCR 扩增反应程序，将 PCR 反应板放置于 PCR 仪上，启动程序。

94 ℃	5 min	
94 ℃	20 sec	45 cycles
56 ℃	30 sec	
72 ℃	1 min	
72 ℃	3 min	
4℃	∞	

2. 产物碱性磷酸酶处理

1) 在 PCR 反应结束后 ,将 PCR 产物用 SAP(shrimp alkaline phosphatase,虾碱性磷酸酶) 处理，以去除体系中游离的 dNTPs。

2) 在新 1.5mlEP 管中配制碱性磷酸酶处理反应液，SAP Mix 反应组分见（表 2）:

表 2. SAP 反应组分

SAP mix of Reagent	Concentration	Volume (1rxn)
Water (HPLC grade)	NA	1.53μl
SAP Buffer	10x	0.17μl
SAP Enzyme	1U/μl	0.30μl
Total volume	-	2.00μl

3) 将 SAP mix 加入 384 孔 PCR 反应板，对于每个碱性磷酸酶处理反应孔，反应总体积为

7μl，其中 PCR 产物 5 μl，SAP mix 2 μl。

4) 移液完成后，小心盖上 384 孔封板膜，并压牢每个孔，防止 PCR 程序时出现蒸发等现象，离心后进行如下反应程序。

5) 设置 SAP 反应程序：37℃ 20min； 85℃ 5min ；4℃ ∞。并将 384 孔反应板放置于 PCR 仪上，启动程序。

3. 单碱基延伸反应

1) 在碱性磷酸酶处理结束后，进行单碱基延伸反应，反应体系总体积 9μl。

2) 在新 1.5mlEP 管中配制单碱基延伸反应液，EXTEND Mix 反应组分见(表 3)：

表 3.延伸反应组分

EXTEND Mix of Reagent	Conc.in 9μl	Volume (1rxm)
Water (HPLC grade)	NA	0.619μl
iPLEX Buffer Plus	0.222x	0.200μl
iPLEX Termination mix	1x	0.200μl
Primer Mix (7 μM: 14 μM)	0.625 uM: 1.25 uM	0.940μl
iPLEX Enzyme	1x	0.041μl
Volume	-	2μl
(7 μM: 14 μM) illustrate the doubled concentration of the high mass primers. Low mass，primers shoμld be at 0.625 uM and high mass primers at 1.25 μM in the final 9 μL reaction.		

3) 将 EXTEND Mix 对应加入 384 孔反应板。对于每个反应孔，单碱基延伸反应体系如下表：

Reagents	Volume (μl)
EXTEND Mix	2

SAP+PCR reaction	7
Total Volume [μl]	9

4) 移液完成后，小心盖上 384 孔封板膜，并压牢每个孔，防止 PCR 程序时出现蒸发等现象，离心后进行如下反应程序。

5) 设置延伸反应程序：

<table>
<tr><td>94 ℃</td><td>30 sec</td><td></td><td></td></tr>
<tr><td>94 ℃</td><td>5 sec</td><td></td><td rowspan="3">40 cycles</td></tr>
<tr><td>52 ℃</td><td>5 sec</td><td rowspan="2">5 cycles</td></tr>
<tr><td>80 ℃</td><td>5 sec</td></tr>
<tr><td>72 ℃</td><td>3 min</td><td></td><td></td></tr>
<tr><td>4℃</td><td>∞</td><td></td><td></td></tr>
</table>

4. 树脂纯化

1) 在 384/6 MG Dimple 板里均匀填充树脂并放置 10 分钟使其晾干。

2) 在 384 样本板的每个孔中加 16 μL 水。

3) 将 384 样本板轻轻翻转过来扣在 Dimple 板上，然后轻敲使树脂落入样本板的每个孔中。

4) 将 384 样本板放置翻转离心机中室温旋转混匀 30 分钟 。

5. 芯片点样

启动 MassARRAY Nanodispenser RS1000 点样仪，将树脂纯化后的延伸产物移至 384-well SpectroCHIP bioarray 上。

6. 质谱检测及数据输出

将点样后的 SpectroCHIP 芯片使用 MALDI-TOF 质谱仪分析，检测结果使用 TYPER4.0 软件获取原始数据及基因分型图，检查数据文件的完整性和正确性，将结果保存入相应存储媒介并递交生物信息室分析。

3.3 数据分析流程说明

1. 单个 SNP 关联分析：通过 Pearson 卡方检验或 Fisher 精确检验，分析正常组和疾病组在每个位点上基因型和等位基因的差异，寻找与疾病相关的位点。
2. LD(linkage disequilibrium)分析：在某一群体中，不同座位上某两个等位基因出现在同一条单体型上的频率与预期的随机频率之间存在明显差异的现象，称连锁不平衡(linkage disequilibrium)。这种不同基因座位的某些等位基因非随机联合经常会在一起遗传。通过 D'/r^2 等考察位点之间的连锁不平衡。
3. 单体型分析：相邻 SNP 的等位位点倾向于以一个整体遗传给后代，位于染色体上某一区域的一组相关联的 SNP 等位位点被称作单体型（haplotype)。通过 Pearson 卡方检验考察这些整体遗传的体型是否和疾病关联。

3.4 SNP genotype 分析方法介绍

1. 统计分析：

基因型频率和等位基因频率计算，并且检验 Hardy-Weinberg 平衡和 MAF，通过 Person 卡方检验比较病例组和对照组之间基因型和等位基因分布情况。

2. 具体分为三种分析：

   a) 单个 SNP 关联分析：通过 Pearson 卡方检验或 Fisher 精确检验，分析正常组和疾病组在每个位点上基因型和等位基因的差异，寻找与疾病相关的位点。
   b) LD(linkage disequilibrium)分析：在某一群体中，不同座位上某两个等位基因出现在同一条单体型上的频率与预期的随机频率之间存在明显差异的现象，称连锁不平衡 (linkage disequilibrium)。这种不同基因座位的某些等位基因非随机联合经常会在一起遗传。通过 D'/r^2 等考察位点之间的连锁不平衡。

c) 单体型分析：相邻 SNP 的等位位点倾向于以一个整体遗传给后代，位于染色体上某一区域的一组相关联的 SNP 等位位点被称作单体型（haplotype)。通过 Pearson 卡方检验考察这些整体遗传的体型是否和疾病关联。

4 附录 实验仪器和设备、试剂或软件

1. 主要实验仪器和设备

仪器名称	仪器来源
低速离心机	湖南湘仪实验室仪器开发有限公司
振荡器	海门市其林贝尔仪器制造有限公司
QB-206 摇床	海门市其林贝尔仪器制造有限公司
电泳槽	北京市六一仪器厂
电子天平	常熟市佳衡天平仪器有限公司
-20℃冰柜	浙江星星家电有限公司
-80℃冰柜	美菱公司
0.1–2.5 μl、0.5–50 μl、50-200 μl、200–1000 μl 移液器	Eppendorf，German
0.5-10 μl 、 20-200 μl 、 100-1000 μl Tip	Axygen 产品
0.5ml、1.5ml 离心管	Axygen 产品
96 孔板、84 孔板	Axygen 产品
96 孔、384 孔 PCR 板胶垫	ABI
核酸自动提取仪	BioTeKe Corpration
NanoDrop2000	Thermo Fisher，USA

仪器名称	仪器来源
Smart view pro 1100 凝胶电泳成像仪	Major science
MP-300V 电泳仪	Major science
ABI veriti-384 PCR 仪	ABI
384-well SpectroCHIP® bioarray 芯片	Agena, Inc
MassARRAY Nanodispenser 点样机	Agena, Inc
MassARRAY Analyzer 4.0 质谱仪	Agena, Inc

2. 主要试剂或软件

试剂或软件名称	仪器来源
DNA 提取试剂盒	BioTeKe Corpration
DNA 染料	BioTeKe Corpration
琼脂糖	BIOWEST,REGMLAR (AGAROSE，G-10)
50×TAE Buffer	Biomed Corpration
6×Loading Buffer	TAKARA BIO INC
ddH_2O	ABI
扩增引物和延伸引物混合物	Thermo
10×PCR 缓冲液	Agena, Inc
15 mM 和 25 mM $MgCl_2$	Agena, Inc
25 mM dNTP 混合物	Agena, Inc
HotStar Taq(5U/μl)	Agena, Inc
SAP Buffer	Agena, Inc

试剂或软件名称	仪器来源
SAP Enzyme	Agena, Inc
iPLEX Buffer Plus	Agena, Inc
iPLEX Termination mix	Agena, Inc
iPLEX Enzyme	Agena, Inc
其他化学试剂	国产分析纯
MassARRAY TYPER4.0	Agena, Inc
