# Supplementary figures and images for: Sporadic Parkinson’s Disease Potential Risk Loci Identified in Han Ancestry of Chinese Mainland
Source: Front Aging Neurosci. 2021 Jan 12;12:603793. doi: 10.3389/fnagi.2020.603793 (PMC7835639; doi:10.3389/fnagi.2020.603793)

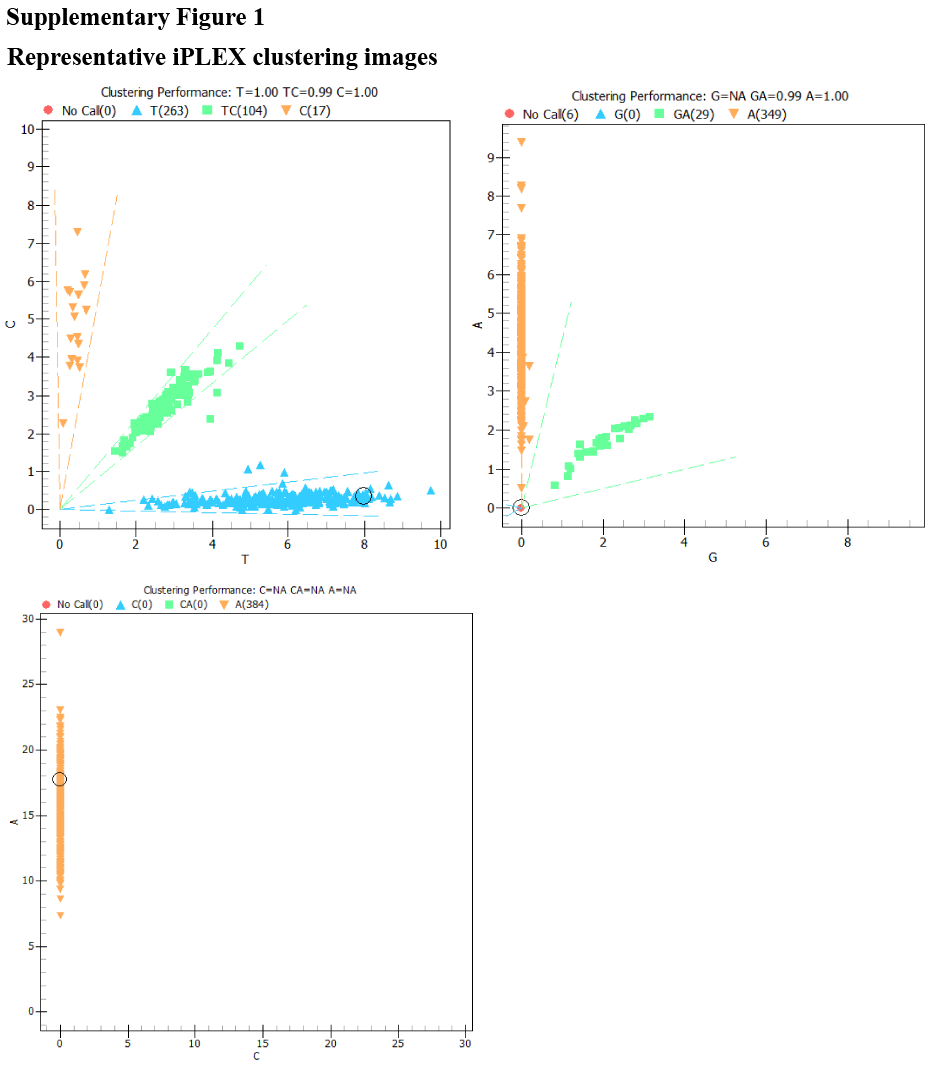

Supplement: Supplementary file 4 [file Image_1.TIF]
